# Response of human macrophages to gamma radiation is mediated via expression of endogenous retroviruses

**DOI:** 10.1371/journal.ppat.1009305

**Published:** 2021-02-08

**Authors:** Natallia Mikhalkevich, Ina P. O’Carroll, Rok Tkavc, Kateryna Lund, Gauthaman Sukumar, Clifton L. Dalgard, Kory R. Johnson, Wenxue Li, Tongguang Wang, Avindra Nath, Sergey Iordanskiy

**Affiliations:** 1 Department of Pharmacology & Molecular Therapeutics, Uniformed Services University of the Health Sciences, Bethesda, Maryland, United States of America; 2 Department of Chemistry, United States Naval Academy, Annapolis, Maryland, United States of America; 3 The Henry M. Jackson Foundation for the Advancement of Military Medicine, Bethesda, Maryland, United States of America; 4 Biomedical Instrumentation Center, Uniformed Services University of the Health Sciences, Bethesda, Maryland, United States of America; 5 The American Genome Center (TAGC), Collaborative Health Initiative Research Program, Uniformed Services University of the Health Sciences, Bethesda, Maryland, United States of America; 6 Department of Anatomy, Physiology & Genetics, Uniformed Services University of the Health Sciences, Bethesda, Maryland, United States of America; 7 Bioinformatics Section, National Institute of Neurological Disorders and Stroke, National Institutes of Health, Bethesda, Maryland, United States of America; 8 Section of Infections of the Nervous System, National Institute of Neurological Disorders and Stroke, National Institutes of Health, Bethesda, Maryland, United States of America; Boston College, UNITED STATES

## Abstract

Ionizing radiation-induced tissue damage recruits monocytes into the exposed area where they are differentiated to macrophages. These implement phagocytic removal of dying cells and elicit an acute inflammatory response, but can also facilitate tumorigenesis due to production of anti-inflammatory cytokines. Using primary human monocyte-derived macrophages (MDMs) and the THP1 monocytic cell line, we demonstrate that gamma radiation triggers monocyte differentiation toward the macrophage phenotype with increased expression of type I interferons (IFN-I) and both pro- and anti-inflammatory macrophage activation markers. We found that these changes correlate with significantly upregulated expression of 622 retroelements from various groups, particularly of several clades of human endogenous retroviruses (HERVs). Elevated transcription was detected in both sense and antisense directions in the HERV subgroups tested, including the most genetically homogeneous clade HML-2. The level of antisense transcription was three- to five-fold higher than of the sense strand levels. Using a proximity ligation assay and immunoprecipitation followed by RNA quantification, we identified an increased amount of the dsRNA receptors MDA-5 and TLR3 bound to an equivalent number of copies of sense and antisense chains of HERVK HML-2 RNA. This binding triggered MAVS-associated signaling pathways resulting in increased expression of IFN-I and inflammation related genes that enhanced the cumulative inflammatory effect of radiation-induced senescence. HML-2 knockdown was accompanied with reduced expression and secretion of IFNα, pro-inflammatory (IL-1β, IL-6, CCL2, CCL3, CCL8, and CCL20) and anti-inflammatory (IL10) modulators in irradiated monocytes and MDMs. Taken together, our data indicate that radiation stress-induced HERV expression enhances the IFN-I and cytokine response and results in increased levels of pro-inflammatory modulators along with expression of anti-inflammatory factors associated with the macrophage tumorigenic phenotype.

## Author summary

Ionizing radiation is a powerful stressogenic factor that induces massive cell damage. The signals released from radiation-damaged tissues recruit the monocytes, which are differentiated into macrophages that remove dying cells via phagocytosis and facilitate inflammation but can also contribute to tumorigenesis through anti-inflammatory and regenerative activities. The mechanism of this dual response of macrophages to irradiation is not fully understood. Using primary human macrophages and a monocytic cell line, we demonstrated that gamma radiation doses activate expression of various human endogenous retroviruses (HERVs). At the molecular level, we have shown that increased numbers of sense and antisense transcripts of tested HERV subgroups bind to double-stranded RNA receptors inducing the expression of type I interferons, multiple pro-inflammatory and some anti-inflammatory factors. At the phenotypic level, polarized macrophages exhibit a potent inflammatory response along with potentially tumorigenic characteristics. Our data suggest that endogenous retroviruses represent an important contributor of the macrophage-mediated inflammation in response to radiation-induced stress but may also indirectly influence tumorigenesis via biased macrophage polarization.

## Introduction

Ionizing radiation (IR) induces cell damage via production of reactive oxygen species (ROS) and induction of DNA breaks. The altered microenvironment leads to changes in the expression of inflammatory cytokines, chemokines, and fibrotic cytokines, in cell–cell interactions, influx of inflammatory cells, and in induction of the reparative and restorative processes [1]. Among the immune cells recruited by the signals released from radiation-damaged tissues, the monocytes play a critical role: they are differentiated into macrophages that remove dying cells via phagocytosis and contribute to induction of the tolerance or stimulation of adaptive antitumor immunity [2–4]. Once differentiated, macrophages can be polarized either into *1*) two functional phenotypes characterized by pro- or anti-inflammatory activity, resulting in contrasting effects on tumor development [5,6], or *2*) a complex or intermediate phenotype with both pro- and anti-inflammatory features [4,7]. Macrophages, particularly the anti-inflammatory ones [8], exhibit increased resistance to IR compared to most other immune cells [4,9,10]. The IR-activated monocyte-derived macrophages (MDMs), along with promotion of acute inflammation, also contribute to cancer cell invasion, proliferation and cancer cell-induced angiogenesis, hence facilitating the development of secondary, radiation-induced cancer [7,9,11–13].

The expression of inflammation-related genes is controlled at multiple levels, including transcription, mRNA processing, translation, phosphorylation, and degradation [14]. Various non-coding RNAs (ncRNAs) and cytoplasmic non-methylated DNA are considered key regulators of inflammation-related gene expression [14–16]. Gene expression changes during macrophage polarization are accompanied by altered long ncRNA and mRNA expression profiles [17]. Intracellular RNA-recognizing toll-like receptors (TLRs), located in the lumen of endosomes, are stimulated by single-stranded (TLR7 and 8) or double-stranded (TLR3) RNA, and induce signaling pathways resulting in activation of innate immunity [18–20]. We previously demonstrated activation of the TLR3 signaling pathway in human macrophages by the binding of the HIV-1 TAR ncRNA to TLR3 [21]. Unlike TLRs, the soluble cytosolic RNA sensors RIG-I and MDA-5 are activated by oligomerization through both RNA- and polyubiquitin-dependent mechanisms [22]. Upon activation, they recruit mitochondrial antiviral-signaling (MAVS) protein and downstream signaling molecules, leading to the activation of the transcription factors IRF3/7 and NF-κB [23]. Both groups of RNA receptors upregulate a set of genes for inflammatory mediators and type I interferons (IFN-I)–IFNα and IFNβ [15,24–26]. While IFN-I are best known for antiviral immune activation through the induction of interferon-stimulated genes (ISG), they are also involved in initiation of senescence via activation of the p53 pathway [27,28], immunosuppression and anti-inflammatory effects through the production of IL-10 and programmed death-ligand 1 (PD-L1) [29–34].

Human Endogenous Retroviruses (HERVs) constitute about 8.3% of the human genome and are considered a potential intracellular source of viral RNA and ssDNA in both normal and pathogenic states [16,18]. Although the formation of infectious particles has not been shown for HERVs and many have inactivating mutations, they are associated with the pathogenesis of multiple types of malignancies, autoimmune and neurodegenerative diseases, including lupus erythematosus, osteoarthritis, ALS, multiple sclerosis, schizophrenia and HIV-associated neurocognitive disorders [35,36]. Multiple reports indicate that HERVs are implicated in both the pathogenic and normal innate immune functions and viral RNA plays an essential role in these responses [18,37–42].

In this study, we investigate the polarization and cytokine response of monocytes and MDMs to therapeutically relevant doses of gamma radiation (_γ_IR) and the role of endogenous retroviruses in these changes. We show that different HERV clades display elevated transcription that in turn facilitates IFN-I and cytokine expression via cytoplasmic RNA receptors MDA-5 and TLR3 and activation of downstream MAVS-mediated pathway. This suggests that HERV expression promotes transition of the irradiated macrophages towards an altered secretory phenotype, which is characterized by increased levels of pro-inflammatory factors, such as IL-6, IL-1β, TNFα, CCL2, CCL3, CCL8, and CCL20, while at the same time the elevated secretion of anti-inflammatory IL-10, which may facilitate tumorigenic activity of these cells.

## Results

### Gamma radiation triggers expression of pro- and anti-inflammatory genes in monocytes and monocyte-derived macrophages

**Fig 1:**
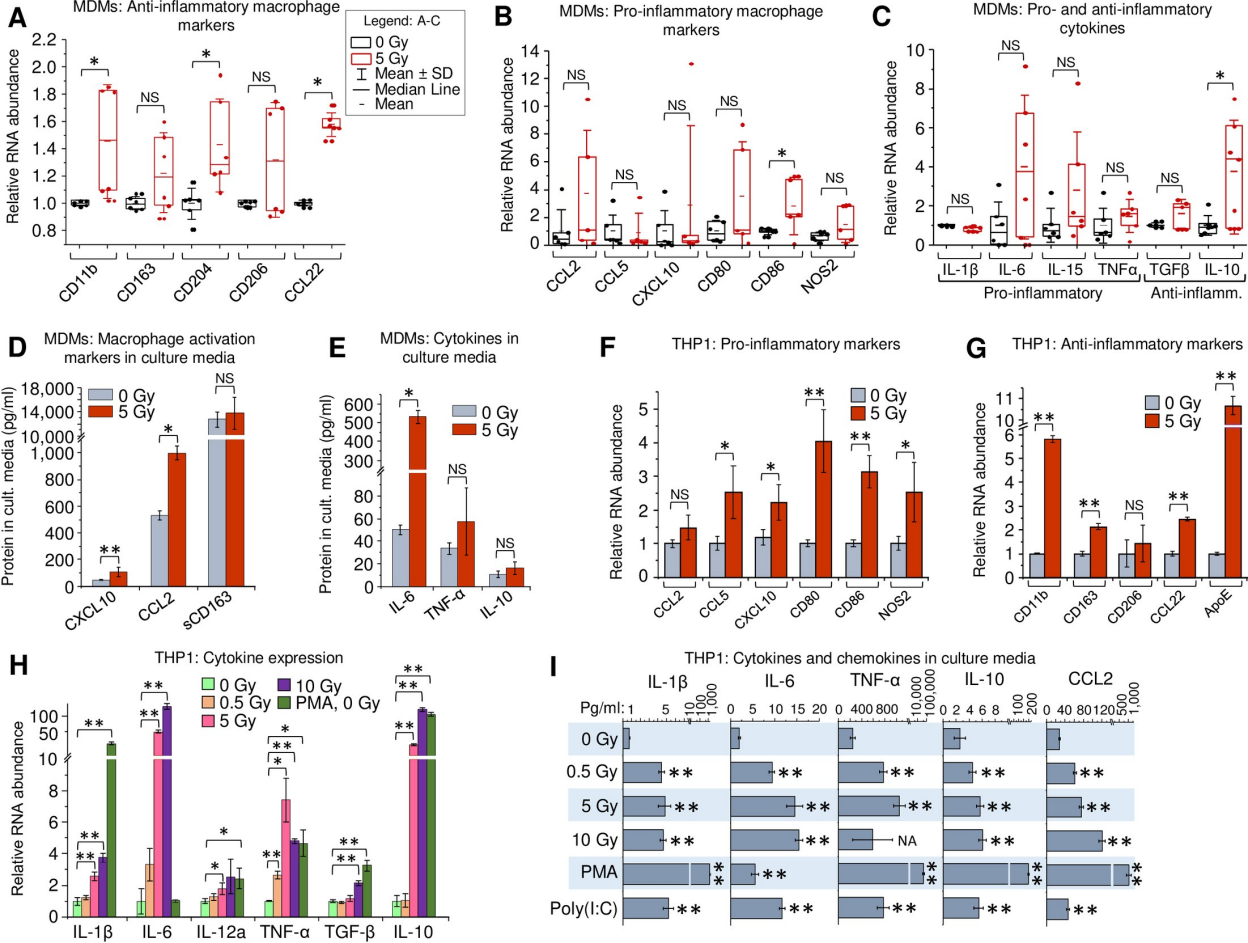
Gamma radiation enhances expression of activation markers in primary human MDMs and THP1 monocytes. (**A-C**) Expression of macrophage anti-inflammatory markers (**A**), pro-inflammatory markers (**B**), and major cytokines (**C**) in primary human MDMs, 48h post-irradiation. MDMs were differentiated from PBMCs, isolated from 6 donors, by treatment with M-CSF and exposed to 5Gy _γ_IR; box plots of RT-qPCR measured expression of n = 6 is shown. * *p*<0.05, paired Wilcoxon test. (**D** and **E**) Protein concentrations of soluble macrophage activation markers CXCL10, CCL2 and sCD163 (**D**) and cytokines IL-6, TNF-α, and IL-10 (**E**) in culture medium of primary MDMs, 48h post-irradiation. Error bars indicate ±SD of three independent biological replicates; * *p*<0.05, ** *p*<0.01, panel D and below–paired *t* test. (**F** and **G**) Expression of macrophage pro-inflammatory (**F**) and anti-inflammatory (**G**) markers in human monocytic cells THP1 48h after exposure to _γ_IR. Error bars indicate ±SD of four independent biological replicates. (**H**) Expression of cytokines in THP1 cells, 48h after _γ_IR. Cells were exposed to indicated _γ_IR doses; RT-qPCR analysis was performed with primers specific for indicated cytokines. Error bars indicate ±SD of 4 independent biological replicates. (**I**) Quantitation of proinflammatory (IL-1β, IL-6, TNF-α, CCL2) and anti-inflammatory (IL-10) cytokines in culture media of _γ_IR-exposed THP1 cells using Luminex multiplex immunoassay, 48h after _γ_IR, PMA (20 nM) or poly(I:C) (2 μg/ml) treatment. Error bars indicate ±SD of three independent biological replicates; ** *p*<0.01 vs 0 Gy. For panels F to I: * *p*<0.05, ** *p*<0.01, ^NS^ non-significant.

To elucidate the impact of _γ_IR on macrophage polarization, we analyzed expression of pro- and anti-inflammatory markers in viable primary human MDMs, derived from peripheral blood mononuclear cells by treatment with macrophage colony-stimulating factor (M-CSF). First, the gene expression was quantitated at 48 h post-exposure to a single 5 Gy _γ_IR dose by RT-quantitative PCR (RT-qPCR). Since _γ_IR can alter expression of many genes, we tested the stability of expression of three housekeeping genes commonly used as reference in RT-qPCR, in radiation-exposed and unexposed MDMs as described earlier [43]. Based on an analysis of at least six biological replicates, we selected β-actin as the most stably expressed reference gene (**S1A Fig**). The M-CSF-treated macrophages exhibit an anti-inflammatory phenotype [44,45]. Interestingly, exposure to _γ_IR led to further increase of expression of many anti-inflammatory markers, such as surface receptors CD11b, CD204, and chemokine CCL22 (**Fig 1A**). Despite a high heterogeneity of macrophage populations, pointed out in many previous studies [46–48], a trend for elevated expression of distinct markers of inflammation, such as CCL2, CD80, CD86 and NOS2 was detected (**Fig 1B**). Analysis of cytokine expression showed a similar result: while only anti-inflammatory IL-10 was significantly increased, the anti-inflammatory cytokine TGFβ and pro-inflammatory IL-6, IL-15 and TNF-α demonstrated an upward trend (**Fig 1C**). Multiplex immunoassay for soluble macrophage markers revealed significantly increased levels of chemokines CXCL10 and CCL2 after IR (**Fig 1D**). Association with macrophage inflammatory responses has been demonstrated for both chemokines [49,50]. A similar upward trend was observed for cytokine secretion in culture media, with IL-6 exhibiting a tenfold increase upon irradiation (**Fig 1E**). Despite the observed diversity in expression of particular macrophage markers among MDM samples, exposure to _γ_IR resulted in a visible trend of upregulated expression of both pro- and anti-inflammatory factors.

To analyze the effect of _γ_IR on monocytes and MDMs in a more homogeneous cell population, we examined the expression of the same genes after _γ_IR in the well-studied human monocytic cell line, THP1. These cells acquire macrophage biochemical and morphological characteristics after treatment with PMA, and are considered a pertinent model of macrophage differentiation [51–53]. The effect of radiation was examined on four housekeeping genes with β-actin again present at highest amount with non-significant deviation upon irradiation (**S1B Fig**). Expression analysis of the macrophage activation markers in irradiated THP1 ‘monocytes’, i.e. in the non PMA-treated cells, revealed significantly increased transcription of inflammation-related chemokines CCL5 and CXCL10, surface receptors CD80 and CD86, and intracellular marker of inflammation NOS2 (**Fig 1F**). Transcription of all the tested anti-inflammatory markers, except CD204 which was undetectable in THP1, was also increased (**Fig 1G**), as was observed in primary MDMs. Evaluation of the surface markers by flow cytometry showed increase of inflammation-related CD80 and CD86, proportional to their RNA level, along with another common marker of macrophage inflammatory response, HLA-DR [4,54] (**S1C**–**S1E Fig**). Macrophage anti-inflammatory marker CD206 [55] also showed an upward trend accordantly with its mRNA level. (**S1F Fig**).

Analysis of cytokine gene expression in irradiated THP1 cells revealed a pattern of increased pro- and anti-inflammatory markers, like that detected in primary MDMs (**Fig 1H**). While the low γIR dose 0.5 Gy did not remarkably affect transcription of the tested genes, exposure to 5 and 10 Gy doses led to significantly elevated expression of IL-1β, IL-6, TNF-α and IL-10, that was the most responsive to IR, displaying 20-100-fold increased transcription. Immunoassay of secreted factors revealed five- to tenfold increase of pro-inflammatory IL-1β, IL-6, TNF-α and CCL2, and two-to-threefold increase of IL-10 in supernatants from irradiated cells (**Fig 1I**).

Treatment of THP1 cells with PMA led to a dramatic increase of all the tested markers except IL-6 (**Fig 1H** and **1I**), indicating that PMA triggers differentiation of THP1 ‘monocytes’ into ‘macrophages’ alongside with their activation to mostly pro-inflammatory secretory phenotype, identically to GM-CSF-induced differentiation of primary monocytes [56]. While PMA-induced differentiation of THP1 toward macrophages is regulated by a complex mechanism associated with expression of several cell cycle regulators [53], our data suggest that PMA and radiation employ different routes to macrophage activation. On the other hand, the treatment with poly(I:C), a synthetic dsRNA analogue commonly used for activation of dsRNA-induced IFN-I expression [20] led to increased levels of all tested cytokines, as was observed in the irradiated cells (**Fig 1I**). This suggests that the cytokine profile of irradiated cells may be related, at least in part, to accumulation of dsRNA and subsequent IFN-I activation.

### Macrophage inflammatory response is related to radiation-induced senescence

**Fig 2:**
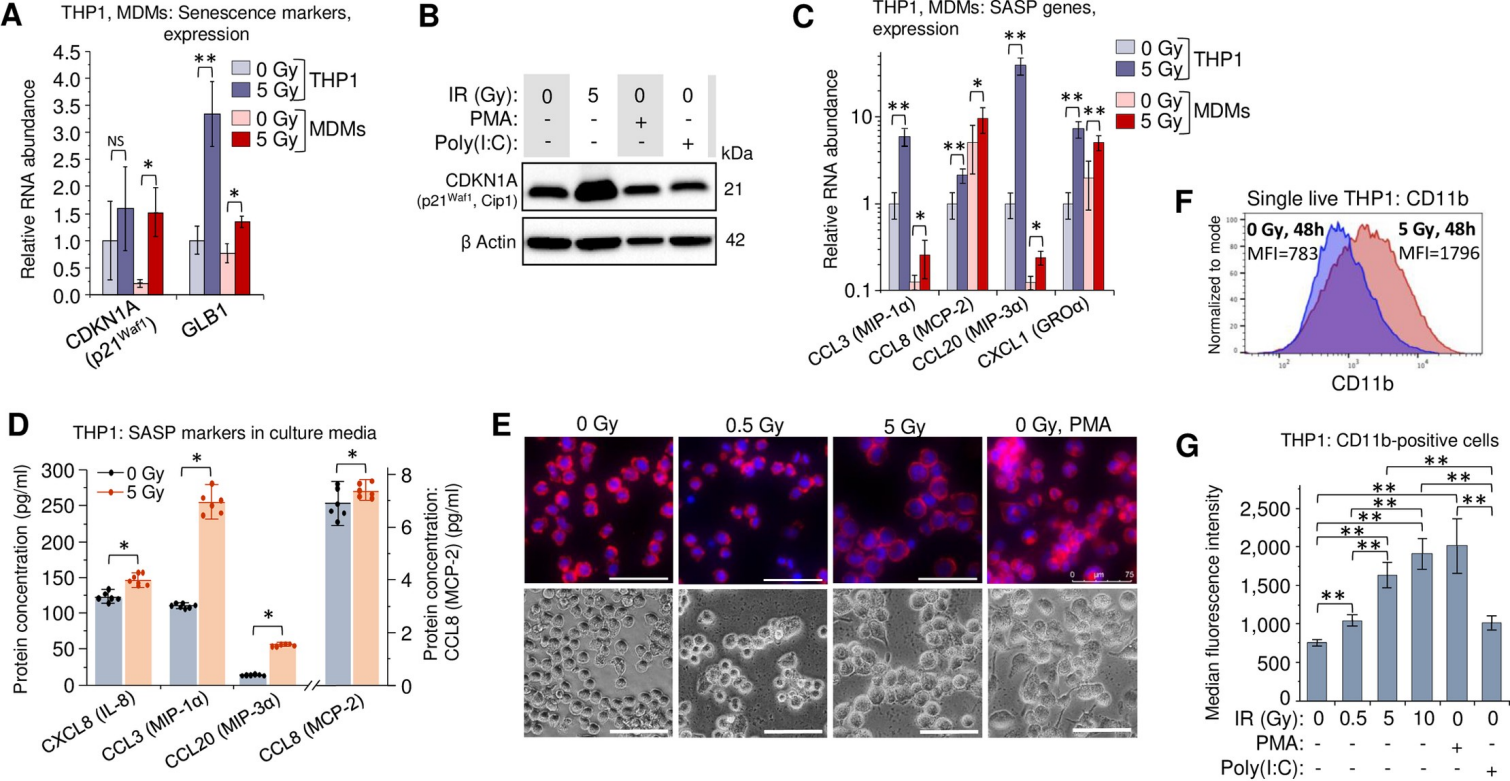
Secretory profile and macrophage phenotype of γIR-exposed monocytes and MDMs are related in part to radiation-induced senescence. (**A**) Expression of intracellular senescence markers (CDKN1A and GLB1) in THP1 cells (blue columns) and primary MDMs (red columns) 48h after irradiation. Error bars: ±SD of three independent biological replicates. (**B**) Western blot analysis of senescence marker p21^Waf1^ (CDKN1A) protein in THP1 lysates, 48 h after irradiation or treatment with PMA or poly(I:C). Forty μg of total protein were loaded. (**C**) Expression of indicated macrophage SASP markers in human monocytic cells THP1 (blue columns) and primary MDMs (red columns) 48h after irradiation. Error bars indicate ±SD of three independent biological replicates. In all panels: * *p*<0.05, ** *p*<0.01, ^NS^ non-significant. (**D**) Quantitation of indicated macrophage SASP markers in culture media of irradiated THP1 cells (n = 6) using Luminex multiplex immunoassay, 48h after γIR. Error bars indicate ±SD; * *p*<0.05, paired Wilcoxon test. (**E**) Microscope images of untreated, irradiated, and PMA-treated THP1 monocytes, 48h incubation. Cells were fixed with paraformaldehyde, labeled for F actin with phalloidin-rhodamine conjugate and for nuclear DNA with DAPI (upper panels) or vital images were obtained (lower panels). Scale bars: 75 μm. (**F**) Proportion of CD11b-positive THP1 cells in irradiated or control cells detected by flow cytometry of viable (DAPI^-^) cells, 72h after _γ_IR (graphs represent median fluorescence intensity data of 4 independent experiments). (**G**) Quantitation (flow cytometry) of CD11b-positive populations in cells exposed to increasing doses of _γ_IR, or treated with PMA or poly(I:C), 72h post-exposure (only viable, DAPI^-^ cells were analyzed; box plot of n = 4 is shown. ** *p*<0.01.

Radiation related DNA damage and oxidative stress cause a cell cycle arrest and induce senescence in the exposed cells. Senescence-associated secretory phenotype (SASP) factors reduce proliferative potential, enhance resistance to apoptosis and alter the immune response of both irradiated and unexposed cells [57,58]. Despite its complexity, most of the SASP-released factors have pro-inflammatory activity [59]. Among them, IL-6 is considered a hallmark of SASP, particularly in the myeloid lineages [59–62]. Since IL-6 expression was dramatically increased in irradiated primary MDMs and THP1 cells (**Fig 1C, 1E, 1H** and **1I**), we assessed the expression of two senescence markers: CDKN1A (p21^Waf1^, Cip1), a hallmark of senescence-associated cell cycle arrest [63,64], and GLB1 (*β*-D-galactosidase, *β*-Gal) which accumulates in cells after activation of the senescence program [65,66]. Indeed, in irradiated THP1 and primary MDMs, both genes exhibited elevated transcription (**Fig 2A**). The p21 protein levels in THP1 cells increased after _γ_IR, but were unaffected by the treatment with either PMA or poly(I:C) (**Fig 2B**).

To assess the effect of _γ_IR on SASP, we then tested irradiated cells for other macrophage- and microglia-specific SASP markers, specifically CCL3 (MIP-1α), CCL8 (MCP-2), CCL20 (MIP-3 α), and CXCL1 (GRO α) [60]. All tested genes were upregulated in the irradiated THP1 cells (**Fig 2C**). The basal expression level of CCL8 and CXCL1 chemokines was higher in the M-CSF-induced primary MDMs and was significantly increased after _γ_IR in THP1. The macrophage inflammatory proteins CCL3 (MIP-1α) and CCL20 (MIP-3α) were also upregulated after radiation exposure, even though basal expression was lower than in THP1 cells. Immunoassay of 5-Gy irradiated THP1 cells also revealed statistically significant increase of all four tested chemokines (**Fig 2D**).

Suspension of THP1 monocytes exposed to a 5-Gy _γ_IR dose transformed within 48–72 h to a morphologically heterogeneous cell population seemingly identical to that of the PMA-treated cells: a mixture of adherent and floating cells with increased average size (**Fig 2E**), larger cytoplasm (**S2A Fig**), formation of filopodia and increased cytoplasmic granulation (**S2B** and **S2C Fig**). The irradiated cells had almost twofold higher count of cytoplasmic granules than non-irradiated and 0.5-Gy-treated cells (**S2D Fig**). In addition to stress granule formation due to DNA damage and oxidative stress [67], these characteristics are also indicative of differentiation to a macrophage phenotype [68]. In fact, increased presentation of the adhesion-associated marker CD11b [69–71] in the population of irradiated THP1 cells (**Fig 2F**) and correlation of this value with _γ_IR dose (**Fig 2G**) support this suggestion. The fraction of cells expressing another adherence marker, integrin β1 (CD29) [69], also increased in THP1 cell population proportionally with _γ_IR dose (**S3 Fig**). Together, these data suggest that radiation-induced oxidative stress, driving at least part of the exposed cells to senescence stage, can also be responsible for the differentiation of THP1 monocytes toward a macrophage-like phenotype. However, similar cytokine secretion in _γ_IR-exposed and non-irradiated THP1 ‘monocytes’ treated with the dsRNA IFN-I inducer poly(I:C) allows us to hypothesize that some cellular RNA sources, in particular dsRNA and related RNA signaling-associated genes might also be responsible for radiation stress-induced alteration of the macrophage phenotype.

### Gamma radiation induces expression of type I interferons and inflammation-related genes in monocytes and MDMs via activation of dsRNA receptors

Earlier reports indicated that interferon-regulatory factors (IRFs) and a group of STAT transcription activators are involved in macrophage polarization, particularly in response to radiation exposure [72,73]. One possible mechanism is that the DNA strand breaks caused by IR lead to the release of ssDNA fragments into the cytoplasm, where they activate the STING/TBK1 pathway, which triggers expression of interferons and other IRF-regulated genes [74–76]. Expression of IFN-I genes and secretion of IFNα and IFNβ, which bind the interferon α and β receptor (IFNAR), induce a signaling pathway resulting in STAT1 activity and induction of multiple interferon-stimulated genes (ISGs) [77]. Meanwhile, the role of IFN-I as well as the potential role of RNA in triggering a macrophage response to radiation-induced stress is still largely unknown [73]. We therefore analyzed the effect of _γ_IR on the IFN-I response and expression of inflammation-related genes and involvement of cytoplasmic RNA receptors.

**Fig 3:**
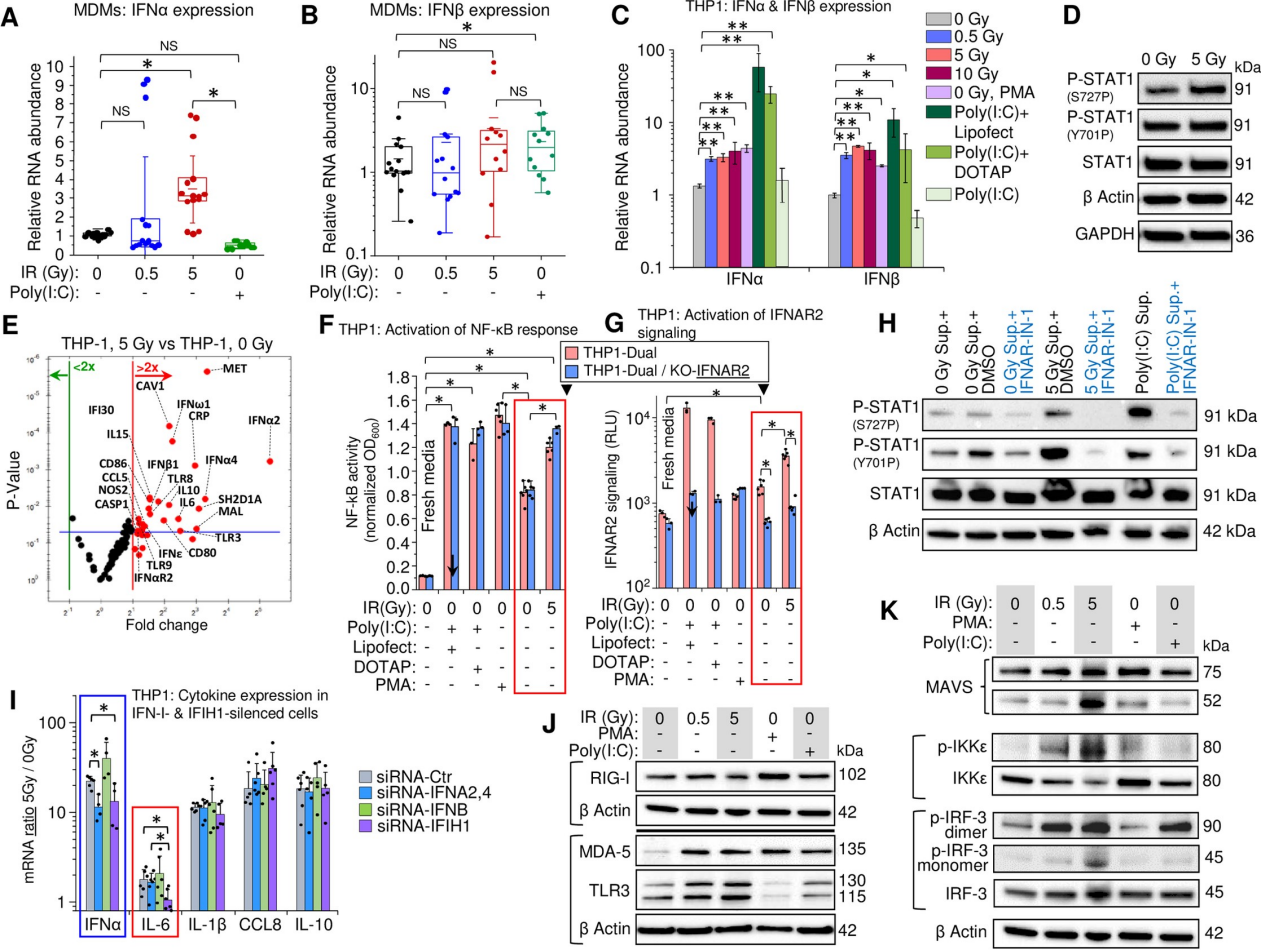
Gamma radiation induces expression of type I interferons and inflammation-related genes in THP1 monocytes and MDMs. (**A** and **B**) Box plots showing expression, measured by RT-qPCR, of IFNα (**A**) and IFNβ (**B**) in MDMs, 48h after irradiation (n = 15). M-CSF-differentiated MDMs from PBMCs isolated from 5 donors were exposed to indicated doses of _γ_IR. Error bars: ±SD, * *p*<0.05, ^NS^ non-significant, paired Wilcoxon test. (**C**) Expression of IFNα and IFNβ in THP1 cells, 48h post-irradiation, measured by RT-qPCR. Cells were exposed to indicated doses of _γ_IR or treated with PMA or poly(I:C) with or without indicated transfection reagents. Error bars: ±SD of four independent biological replicates; * *p*<0.05, ** *p*<0.01. (**D**) Immunoblotting of phosphorylated STAT1 at Ser727 and Tyr701 (40 μg of total protein) in THP1 cells, 48h post-irradiation. (**E**) Volcano plot showing ISG expression in irradiated vs. control THP1 cells, measured by PCR array of total cellular RNA samples, 48h post-irradiation. Red dots indicate significant (above blue line) or insignificant (below blue line) increase in gene expression in irradiated cells (*p*<0.05, fold change >2.0). (**F** and **G**) Activation of NF-κB-dependent transcription (**F**) and IFNAR signaling (**G**) by culture media from irradiated (5 Gy) or non-irradiated THP1 in the reporter THP1-Dual cells (pink columns) expressing SEAP gene driven by an IFN-β minimal promoter activated by NF-κB and secreted Lucia luciferase gene under the control of an IFNAR signaling-activated ISG54 minimal promoter, and THP1-Dual KO-IFNAR2 cells (blue columns), generated from THP1-Dual cells by stable knockout of the IFNAR2 receptor. Similarly, the culture supernatant from Poly(I:C)-transfected THP1 was used as a positive control. After 18 h of incubation and staining with SEAP-sensitive QUANTI-Luc dye, the absorbance (600 nm) (F) or luminescence (G) of each sample were measured and normalized to fresh RPMI media controls. Error bars indicate ±SD of a minimum of 3 independent biological replicates; *p*<0.01. (**H**) Immunoblotting to assess STAT1 Ser727 and Tyr701 phosphorylation (20 μg of total protein) in THP1 cells incubated for 24h with filtered culture media from irradiated or non-irradiated THP1, with or without interferon-α/β receptor inhibitor IFNAR-IN-1. (**I**) Expression ratio of IFNα and indicated cytokines (5 Gy-to-0 Gy ratio of RNA count) in THP1 cells transfected with indicated siRNA (24 h before irradiation), with or without exposure to 5 Gy _γ_IR for 48 hours. RNA was quantified by RT-qPCR. Error bars: ±SD of at least four independent biological replicates; * *p*<0.05. (**J**) Immunoblot of ssRNA sensor RIG-I and dsRNA sensors MDA-5 and TLR3 (40 μg of total protein) in THP1 cells, 48h post-irradiation. (**K**) Western blot of downstream markers of the activation of RIG-I/MDA-5 (MAVS, phospho-IKKε, and phospho-IRF3) and TLR3 (phospho-IKKε, and phospho-IRF3) pathways. THP1 lysates (40 μg total protein), harvested 48h post-exposure.

Despite the high heterogeneity of MDM populations, transcription levels of IFNα were significantly increased 48 h post-exposure to a 5 Gy IR dose (**Fig 3A**) and the median transcription level for IFNβ displayed an upward trend in response to a 5-Gy γIR exposure, though this was not statistically significant (**Fig 3B**). Surprisingly, the dsRNA poly(I:C) induced an INFβ, but not an IFNα response in macrophages. However, when poly(I:C) was transfected into the cells using lipophilic transfection reagents, a dramatic increase of both IFNα and IFNβ transcription was observed even 24 h post-transfection (**Figs 3C** and S4A). The delayed IFN-I response to _γ_IR suggests induction of an internal activator of IFN-I expression, that must accumulate before it exerts a measurable impact.

We then analyzed IFN-I-stimulated STAT1 activation and consequent ISG expression. Phosphorylation of Y701 and S727 residues is critical for STAT1 activity [78,79]. Increased phosphorylation was only observed for S727, which is critical for the transcription activation function of this protein [79] (**Fig 3D**). Accordingly, profiling of ISG expression in THP1 cells displayed increased transcription of multiple genes, including significantly elevated macrophage inflammation markers CD80, CD86, NOS2, IL-6, and IL-15 along with anti-inflammatory IL-10 (**Figs 3E** and S4B). The same analysis of primary MDMs also revealed elevated expression of IL-6, IL-10, and IL-15 after irradiation (**S4C Fig**).

To test involvement of the IFN-I pathway in the observed cytokine elevation upon irradiation, we treated reporter THP1-Dual cells, expressing secreted embryonic alkaline phosphatase gene driven by a recombinant promoter activated by NF-κB and the Lucia luciferase gene activated by signaling from an interferon-α/β receptor (IFNAR). Another engineered cell line, THP1-Dual KO-IFNAR2, generated from THP1-Dual cells by stable knockout of the IFNAR subunit 2, was used as a negative control to test IFN-I stimulated response. Treatment of reporter cells with the culture media from regular THP1 cells irradiated with a 5-Gy dose led to a significant increase of NF-κB-activated expression in both reporter cell lines (**Fig 3F** red frame), suggesting that elevated expression of inflammation-related genes was activated by the signaling pathways beyond IFN-I-induced IFNAR signaling. Interestingly, treatment of both reporter cell lines with the culture media from the poly(I:C)-transfected cells also increased NF-κB-stimulated expression independently of the signaling from interferon receptor. However, analysis of IFNAR signaling-dependent luciferase expression showed at least threefold increase of luciferase activity only in IFNAR-positive cells, whereas the knockout cell line did not respond to the treatment with culture media from either irradiated or poly(I:C)-transfected THP1 (**Fig 3G**). Indeed, treatment of THP1 with the media harvested from irradiated or non-irradiated cells, that contained either interferon-α/β receptor inhibitor (IFNAR-IN-1) or only DMSO diluent, resulted in markedly reduced phosphorylation of both S727 and Y701 in the presence of the inhibitor (**Fig 3H**). Together, these data indicate that elevated expression of inflammation-related genes in human monocytes and macrophages is mediated by various signaling pathways, although the IFN-I signaling also takes part in this response and likely enhances expression of some of these genes.

To check whether IFN-I signaling is involved in the increased expression of particular cytokines after irradiation, we measured their mRNA ratios in 5 Gy γ-irradiated vs. non-irradiated THP1 cells transfected with siRNAs that targeted either type I interferons (IFNA and IFNB genes) or the dsRNA receptor MDA-5 (IFIH1 gene). As expected, transfection of the cells with both siRNA-IFNA2,4 (siRNA mix silencing IFNα2, 4, 7, 10, and 17) and siRNA-IFIH1 resulted in decreased IFNα expression, whereas transfection with siRNA-IFNB did not alter IFNα mRNA levels (**Fig 3I** blue frame). Among the four tested cytokines, IL-6 displayed significantly reduced expression when the cells were transfected with siRNA-IFIH1 (**Fig 3I** red frame), suggesting that expression of this cytokine was dependent on dsRNA signaling through the MDA-5 receptor.

Our data suggest that _γ_IR activates RNA signaling-mediated molecular mechanisms. Indeed, we found that cytoplasmic dsRNA receptors exhibit increased expression in irradiated THP1. Only long-dsRNA sensors, MDA-5 and TLR3, were increased after _γ_IR, whereas RIG-I, the sensor for 5’ triphosphate-ssRNA and short dsRNA duplexes, remained unaffected (**Fig 3J**). Expectedly, treatment with poly(I:C) led to similar results. Analysis of cytoplasmic ssRNA sensors revealed no effect on full-length TLR7 and TLR8 in irradiated cells, while the functionally-competent cleaved form of TLR7 [80] was increased after _γ_IR, and especially PMA treatment, suggesting induction of ssRNA signaling at least in the endosomal compartment (**S4D Fig**).

The signaling pathways induced by binding of RNA to RIG-I/MDA-5 and TLR3 have different initial steps but share the same protein kinases IKKε and IKKβ, that in turn activate TBK1, IRF-3 and NF-κB [81]. A specific marker of RIG-I/MDA-5 signaling, the mitochondrial antiviral-signaling (MAVS) protein [82], was found increased in the cytoplasm of irradiated and PMA-treated THP1 (**Fig 3K**). However, only exposure to a 5-Gy dose dramatically increased MAVS cleavage (52kDa band), shown to be induced by dsRNA during viral infection [83]. The phosphorylated forms of IKKε and IRF-3, markers of both RIG-I/MDA-5 and TLR3 pathways, were also increased dose-dependently upon IR. IRF-3 that activates IFN-I transcription via phosphorylation-dependent dimerization [84] displayed increased number of dimers in THP1 after _γ_IR and poly(I:C) treatment. Together, our data indicate that activation of dsRNA-triggered pathways in irradiated monocytes/macrophages results in the expression of IFN-I as well as multiple inflammation-related genes in both the irradiated and bystander cells.

### Gamma radiation activates transcription of HERV clades in primary MDMs and in the THP1 monocytic cell line

**Fig 4:**
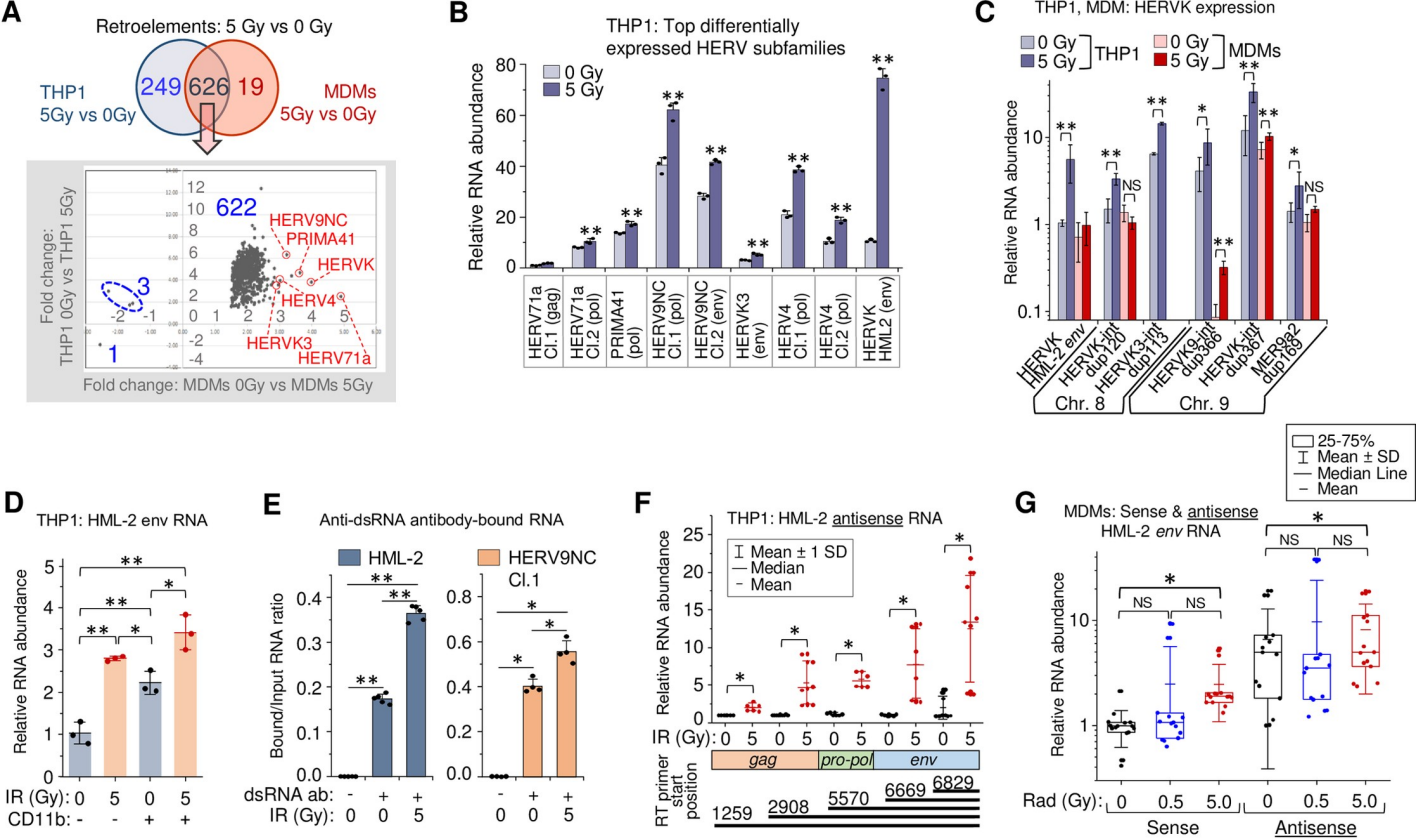
Gamma radiation activates HERV transcription in primary human MDMs and THP1 cells. (**A**) *Top*: Venn diagram of differentially expressed retroelements and HERVs identified between irradiated vs. control THP1 cells or MDMs. *Bottom*: expression fold changes detected for the common set (626 features) shown on the upper Venn diagram. (**B**) Expression of six HERV clades, identified upregulated by RNA-Seq in both irradiated THP1 cells and MDMs (panel A, red circles), measured by RT-qPCR at 48h post-exposure to 5 Gy _γ_IR dose. Primers were designed for the alignments within the clusters identified across *gag*, *pol* or *env* genes of the sequences within the clades. Error bars: ±SD of three independent biological replicates. (**C**) Expression, measured by RT-qPCR, of randomly selected differentially expressed HERVK in THP1 cells (grey columns) and primary MDMs (red columns), 48h post-irradiation. Error bars: ±SD of three independent biological replicates. (**D**) Transcription of HERVK HML-2 *env* in response to 5 Gy _γ_IR dose, measured by RT-qPCR at 48h post-exposure in CD11b-positive and negative THP1 cells, separated by sorting flow cytometry. Only viable, DAPI-negative cells were analyzed; box plot of n = 3 is shown. Error bars: ±SD of three independent biological replicates. (**E**) Ratio of HERV RNA bound to anti-dsRNA antibodies to total RNA input: HML-2 (left) and HERV9NC Cl.1 pol (right) from 5 Gy _γ_IR exposed and unexposed THP1 cells. RT-qPCR of RNA-IP (RIP) complexes with rJ2 and 9D5 antibodies, 48h post-irradiation. Error bars: ±SD of five (HML-2) or four (HERV9NC) independent biological replicates; in panels B-E,* *p*<0.05, ** p<0.01. (**F**) Relative count of HERVK HML-2 antisense RNA identified with antisense strand-specific reverse transcription primers targeting regions in the *env*, *pol* and *gag* genes, and quantitated by qPCR. Error bars: ±SD of at least 6 independent biological replicates. (**G**) Relative count of the sense and antisense transcripts of HERVK HML-2 measured by RT-qPCR using *env-*specific primers, 48h after irradiation of MDMs. MDMs were differentiated with M-CSF from PBMCs isolated from 5 donors. Box plot of n = 15 is shown. In panels F and G, * *p*<0.05, ^NS^ non-significant, paired Wilcoxon test.

To identify the source of RNA that activates IFN-I response and cytokine expression in _γ_IR-exposed THP1 cells and primary MDMs, we assessed transcription of transposable retroelements and endogenous retroviruses via RNA-seq analysis. These were chosen because they are abundant in the genome and activation of nearby genes can result in their transcription in both directions, depending on their orientation in the loci [41,85]. Moreover, expression of different HERV groups has been shown associated with various types of cancer, neurodegenerative and autoimmune diseases [40,42,86–88]. Using the TEToolkit suite developed by the Hammell group (see Materials and Methods), we identified 955 distinct differentially expressed retroelements and ERVs, 875 and 645 of which were differentially expressed upon γIR in THP1 cells and MDMs, respectively (**S5A Fig** and **Panels A and B in S1 Table**). A total of 626 retroelements were expressed differentially in both irradiated THP1 and MDMs (**Fig 4A** and **Panel C in S1 Table**). Almost all the common retrotransposable sequences in monocytes and MDMs were concordantly upregulated upon γIR. To validate the RNA-seq analysis, we performed RT-qPCR on six of the most upregulated HERV clades detected by RNA-seq analysis (red circles in **Fig 4A**). All these subfamilies include multiple heterogeneous proviral sequences. Thus, to determine genome regions suitable for PCR quantitation, we clustered sequences within each selected subfamily using the ClustralW and Jalview multiple sequences alignment tools and then designed primers for the least variable region within each cluster. All the tested clades displayed significantly increased transcription levels in irradiated THP1 ‘monocytes’ (**Fig 4B**). Interestingly, the highest ratio of transcription activation upon irradiation was detected for the *env* gene of HERVK HML-2 subfamily, probably because that region was relatively conservative among the proviral sequences within this evolutionary youngest and genetically homogenous HERV subgroup [89].

To test whether the potentially disease-associated HERV genomes [40,42,86–88] are upregulated by radiation, we performed RT-qPCR with a panel of primers recognizing 43 HERV sequences. We identified 32 proviruses or ¾ of all tested HERVs, which were upregulated upon irradiation (**S5B Fig**). Radiation led to a significant increase of HML-2 *env* RNA. The background transcription level of this gene was also high. However, this RT-qPCR result could, in part, depend on the lower sequence variability of HML-2.

RNA-seq analysis identified 194 differentially expressed HERVK sequences, 74 of which were found upregulated in irradiated THP1 cells and 54 in the MDMs (**Panels D and E in S1 Table**). To evaluate the impact of _γ_IR on particular HERVK subgroups, we quantified the transcription of six randomly selected HERVK proviral sequences localized in chromosomes 8 and 9 by RT-qPCR in THP1 cells and MDMs. Of the six, only HERVK3-int dup113 was shown to be upregulated significantly by RNA-seq. However, expression of all tested proviruses displayed increased transcription in irradiated THP1 (**Fig 4C**), while the data were more variable in MDMs, probably due to a lower heterogeneity of the cell line versus the MDMs collected from different individuals. Together, these data suggest that IR activates transcription of a larger number of HERV loci than was identified by RNA-seq.

The relative homogeneity of the HERVK HML-2 subfamily allows for a more reliable quantitative assessment of its expression. We probed HML-2 *env* and its 5’LTR to assess the transcription level of the coding and noncoding sequences, including solo-LTRs in irradiated THP1. To evaluate the effect of different _γ_IR doses and macrophage differentiation stimuli on HERVK transcription, we exposed the cells to various doses of _γ_IR or treated with PMA. Even though the relative count of 5’LTR RNA was predictably 30-50x higher than *env*, exposure of cells to increasing doses of _γ_IR gradually enhanced the level of both RNAs three- to tenfold during 48 h after IR exposure (**S5C** and **S5D Fig**). PMA treatment also activated HERVK transcription within the first 24 h, but RNA levels did not increase further with time and did not exceed the impact of the 5 Gy _γ_IR dose. To compare _γ_IR-activated HERV expression in ‘monocytes’ versus the subpopulation of THP1 cells differentiated into ‘macrophages’ after _γ_IR exposure, we analyzed HML-2 *env* transcription in irradiated cells that were sorted by the presence of the adhesion marker CD11b, typically upregulated in mature macrophages [69–71]. **Fig 4D** shows that the basic transcription level, as well as the level of radiation-activated transcription was higher in THP1 exhibiting macrophage phenotype. Analysis of transcription dynamics over the 8-day period showed that after the peak at 48 h post-IR, the *env* RNA levels did not change remarkably in the cells exposed to high doses. However, in 0.5 Gy-exposed cells, *env* expression gradually decreased to the initial level (**S5E Fig**). Together, these data suggest that while the differentiation of monocytes towards macrophages itself activates retroviral transcription, _γ_IR appears to be a stronger inducer of HERV activation and has a prolonged effect.

### Gamma radiation induces double-stranded HERV RNA accumulation

Previous work has shown that dsRNA of various HERVs accumulates in cancer cells treated with DNA methyltransferase inhibitors [40]. To test whether irradiation also results in dsRNA formation, we precipitated dsRNA using two anti-dsRNA monoclonal antibodies from the total RNA extracted from radiation-exposed and non-exposed THP1, as described earlier [90]. Subsequent RNA quantitation with HML-2 *env* and HERV9NC *pol* primers revealed increased levels of dsRNA molecules of both HERV subfamilies in irradiated cells (**Fig 4E**). To identify the potential mechanism responsible for elevated retroviral dsRNA in irradiated cells, we examined HERVK antisense transcription. Utilizing a strand-specific RT-qPCR method [91], we found that all HERVK proviruses that displayed increased levels of the positive RNA strand transcription also had elevated count of antisense RNA in irradiated THP1 (**S5F Fig**). Quantification of the transcripts with primers specific for antisense sequences localized in HML-2 *env*, *pol* and *gag* regions showed a similar basic level of all measured transcripts in non-irradiated THP1 (**Fig 4F**). In contrast, exposure to a single 5 Gy dose significantly increased antisense transcription of all these sequences, with shorter (anti-*env*) transcripts detected at the highest level. Comparison of sense and antisense transcription of *env* with PCR primers recognizing the same area in both directions (nt. 7989–8152) displayed a two- to fourfold higher level of basic antisense transcription in non-irradiated THP1 (**S5G Fig**). Moreover, antisense transcription was remarkably increased after 5 Gy _γ_IR within the first 24 h, whereas elevation of sense transcription was delayed.

Similarly, analysis of HML-2 *env* sense and antisense RNA in primary MDMs showed twofold elevated transcription in the sense direction after 5 Gy _γ_IR dose (**Fig 4G**). Interestingly, the median level of antisense *env* RNA in non-irradiated MDMs was fivefold higher than of the sense RNA, probably due to an activating effect of M-CSF on host-cell promoters outside the provirus, which might drive HERV antisense transcription [85]. Exposure to IR did not significantly change the already high antisense RNA levels. Meanwhile, activation of *env* transcription in the sense direction could ultimately lead to an overall increase of dsRNA in irradiated MDMs.

### HERV expression enhances IFN-I and inflammation-related cytokine response and alters macrophage secretory profile

**Fig 5:**
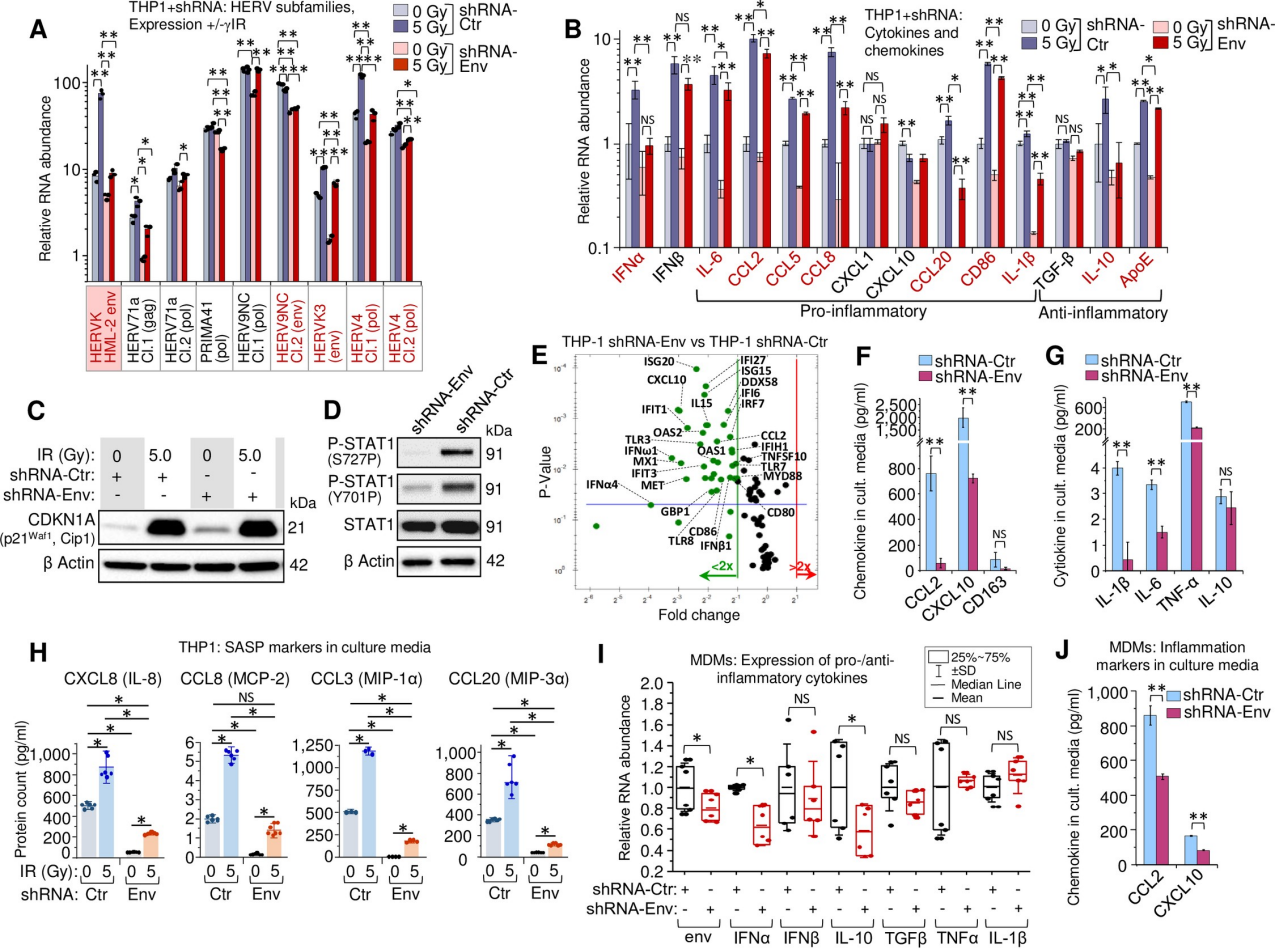
HERVK HML-2 *env* knockdown decreases IFN-I expression and alters the secretory profile of THP1 monocytes and primary MDMs. (**A**) Transcription of HERVK HML-2 and five other HERV clades, identified upregulated by RNA-Seq in both irradiated THP1 cells and MDMs (Fig 4A), measured by RT-qPCR in THP1 cells expressing control (grey) or shRNA-Env (red), 48h post-irradiation. Error bars: ±SD of three independent biological replicates. (**B**) Expression, measured by RT-qPCR, of macrophage activation markers in human monocytic THP1 cells, expressing shRNA-Env (red) or control shRNA (grey), 48h post-irradiation. Error bars: ±SD of three independent biological replicates. In panels A and B, * *p*<0.05, ** *p*<0.01; A and B, genes, whose expression changed significantly in response to shRNA-Env are indicated by red symbols. (**C**) Immunoblot against intracellular senescence marker p21^Waf1^ (CDKN1A) in lysates (40 μg total protein) of THP1 cells, expressing either shRNA-Env or control shRNA, 48h after irradiation. (**D**) Phosphorylation of STAT1 Ser727 and Tyr701 (40 μg total protein) in THP1 cells, expressing control shRNA or shRNA-Env, 48h post-irradiation. (**E**) Volcano plot depicting ISG expression in THP1 cells expressing shRNA-Env or control shRNA, 48h post-IR: measured by PCR array of total cellular RNA samples. Green dots represent significant (above blue line) or non-significant decrease in expression of indicated genes in shRNA-Env vs. control shRNA expressing cells (*p*<0.05, fold change >2.0). (**F** and **G**) Soluble macrophage activation markers (**F**) and key cytokines (**G**) in culture medium of THP1 cells, 48h post-irradiation, determined by multiplex immunoassay (Luminex). Error bars: ±SD of three independent biological replicates. * *p*<0.05, ** *p*<0.01, ^NS^ non-significant. (**H**) Macrophage SASP markers in culture media of irradiated THP1 cells (n = 6), expressing shRNA-Env (yellow) or control shRNA (blue), using multiplex immunoassay (Luminex), 48h after _γ_IR. Error bars indicate ±SD; *p* value of all differences is <0.05, unpaired Mann-Whitney test. (**I**) Transcription levels, measured by RT-qPCR, of HERVK HML-2 *env*, IFN-I (IFNα, IFNβ), inflammation (IL-1β, TNF-α) and anti-inflammatory (TGF-β, IL-10) markers in irradiated primary human MDMs, expressing control or shRNA-Env, 48h post-irradiation. M-CSF-differentiated MDMs from n = 6 donors. * *p*<0.05, ^NS^ non-significant, paired Wilcoxon test. (**J**) Soluble macrophage activation markers in culture medium of primary human MDMs, 48h post-irradiation assessed by multiplex immunoassay. Error bars: ±SD of three independent biological replicates; ** *p*<0.01, two-tailed paired *t* test.

To test whether HERV RNA is involved in activation of the IFN-I response and biased secretory profile of irradiated cells, we developed lentivirus constructs expressing shRNA that targeted less variable regions of HML-2 *env*. To assess the off-target effects of shRNA, we estimated the number of complementary regions with and without mismatches in human mRNA sequences based on the hg38 human genome assembly using GGGenome [92]. Results show that both shRNAs were specific against *env* RNA of multiple HML-2 loci (**Panels A and B in S2 Table**). Heterogeneity of HERV elements in human cells limits the efficiency of RNA silencing. Quantitation of HML-2 RNA showed that both tested shRNAs exerted approximately 50 to 60% knockdown of *env* RNA (**S6A Fig**). In THP1 cells, using a mixture of two *env*-targeting shRNAs (shRNA-Env) remarkably reduced radiation-activated HML-2 transcription (**Fig 5A**, pink box). Surprisingly, several additional HERV clades that have been found upregulated after irradiation, such as HERV9NC, HERVK3, and HERV4 also displayed reduced RNA count in shRNA-expressing cells with and without exposure to _γ_IR (**Fig 5A**). Moreover, the HERVK proviral sequences from chromosomes 8 and 9, tested earlier for their level of transcription after irradiation, also showed a partial knockdown in the shRNA-expressing cells, whereas their transcription was dramatically increased in irradiated cells expressing control shRNA (**S6B Fig**). The mechanism of this indirect effect of HML-2 *env*-targeting shRNA on the transcripts of other HERV subfamilies is not entirely clear, but it can be related to negative feedback events discussed below.

We then asked whether the HML-2 *env*-targeting shRNAs affect accumulation of the viral dsRNA in THP1 cells upon irradiation. Precipitation of dsRNA followed by RT-qPCR to quantitate HML-2 *env* RNA showed marked decrease of this particular dsRNA abundance in both _γ_IR exposed and unexposed cells expressing shRNA-Env (**S6C Fig**).

Analysis of the effect of HERV RNA silencing on the expression profile of the markers of macrophage inflammatory and anti-inflammatory phenotypes revealed dramatically decreased levels of IFNα and approximately 40% decrease of IFNβ transcription in irradiated cells expressing shRNA-Env (**Fig 5B**). Accordantly, the shRNA expression led to significantly reduced transcription of multiple inflammation-related secretory factors, including senescence related IL-6, CCL8, and CCL20 in the irradiated cells. We note that IFN-I activity reduced by HERV knockdown did not alter RNA level of the chemokines CXCL1 and CXCL10: expression of the first one has been shown to be dependent on TLR4 activation [93], whereas the second chemokine is known as IFN_γ_-induced protein (IP-10) [94,95]. Interestingly, among three tested anti-inflammatory genes, TGF-β, IL-10, and ApoE, expression of ApoE, and especially IL-10, was also decreased in the shRNA-Env-positive cells.

Since a few of the inflammatory factors we found suppressed in the irradiated cells upon shRNA-Env expression are the markers of SASP, we checked whether shRNA can alter the senescence response to IR. Immunoblotting of THP1 cell lysates showed increased levels of the P21^Waf1^ protein after IR, independently of HERV-K knockdown (**Fig 5C**). This suggests that radiation-induced expression of some SASP-related factors, such as CCL8 and CCL20, can be enhanced by HERV, possibly via IFN-I activation.

To assess involvement of IFN-I in this response, we analyzed the STAT1 phosphorylation status after HERV-K-knockdown. Both Y701 and S727 were hypophosphorylated in the shRNA-Env-expressing cells (**Fig 5D**), indicating that HERV expression is involved in activation of IFN-I pathway. Indeed, evaluation of ISG transcription by PCR array in irradiated THP1 cells after partial HERV knockdown demonstrated significant inhibition of multiple IFN-induced genes, including macrophage inflammation activation markers, such as CD80, CD86, CCL2, and IL-15 (**Figs 5E** and S6D). Immunoassay of the secreted factors confirmed that while the proinflammatory cytokines IL-1β, IL-6, TNF-α, including chemokines CCL2 and CXCL10, were dramatically decreased in culture media of irradiated THP1 that expressed *env*-targeting shRNA (**Fig 5F** and **5G**), the anti-inflammatory marker CD163 was also reduced. Analysis of SASP markers in culture media revealed the same trend for all tested proteins: *env* partial knockdown significantly reduced secretion of inflammation-related chemokines from both unexposed and γIR-exposed cells (**Fig 5H**).

Gene expression in primary MDMs infected with lentivirus expressing shRNA-Env recapitulated the trend observed in THP1 cells: decreased HML-2 *env* RNA level correlated with suppressed expression of IFNα and IL-10 (**Fig 5I**). At the protein level, partial HERV knockdown resulted in a twofold decrease of inflammation-related chemokines (**Fig 5J**). Taken together, the data suggest that the HERV RNA levels, likely dsRNA, are important for activation of IFN-I, especially IFNα, and multiple markers of innate immune response whose expression might depend on the viral RNA-induced signaling.

### HERV RNA induces type I interferon and cytokine expression via MDA-5 and TLR3 receptors

Several independent research groups have demonstrated HERV involvement in the innate immune response, mediated by cytoplasmic RNA sensors, such as RIG-I/MDA-5 and nucleic acid-binding TLRs [37,40,96–99]. Based on our data that expression of IFNα and multiple cytokines correlated with HERV transcription level and in particular dsRNA in monocytes/macrophages, we hypothesized that viral transcripts directly bind to cytoplasmic RNA sensors and activate downstream signaling pathways.

**Fig 6:**
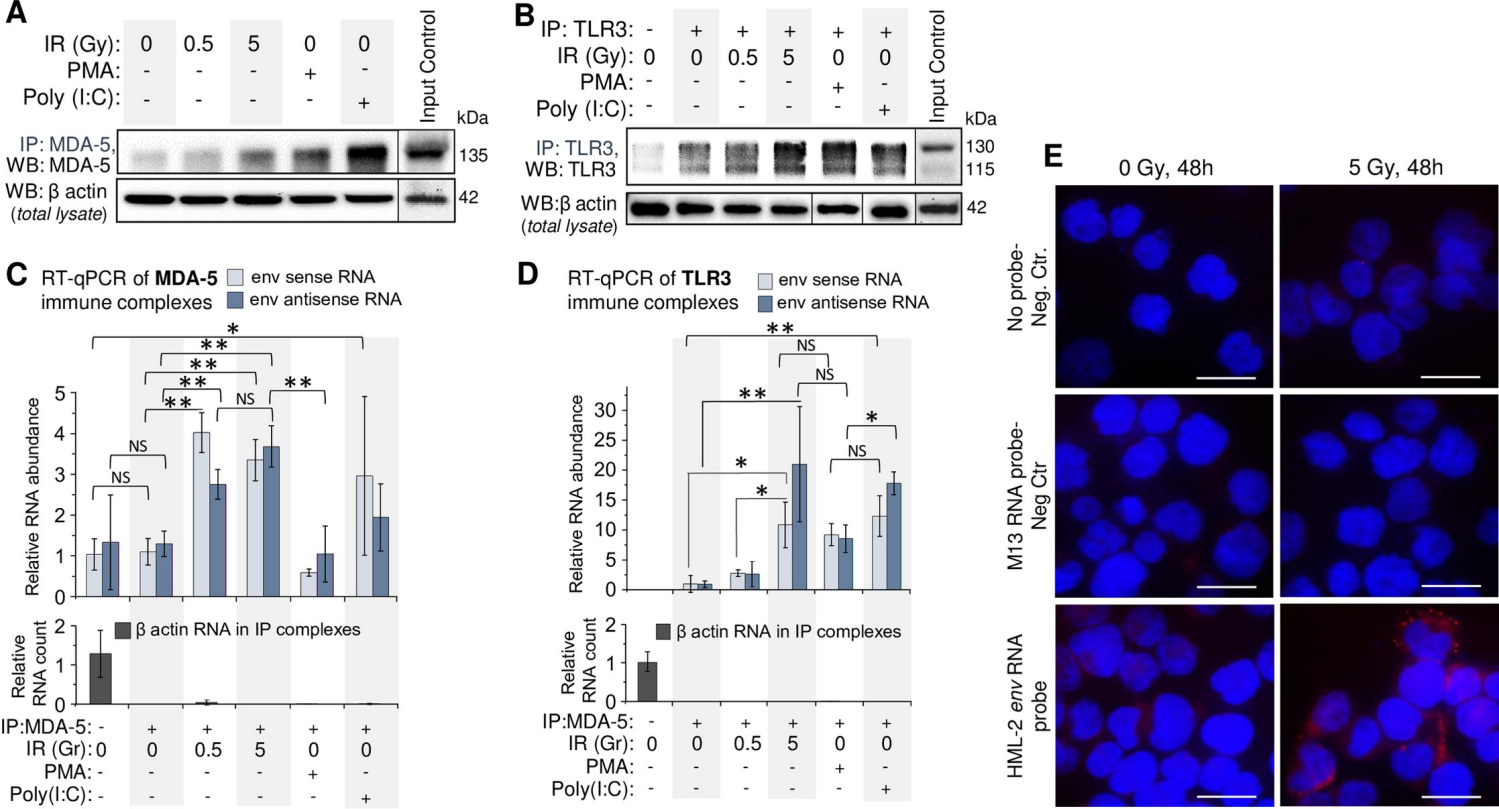
HERVK *env* RNA is associated with cytoplasmic dsRNA receptors MDA-5 and TLR3 in irradiated monocytes THP1. (**A** and **B**) Immunoblot of MDA-5 (**A**) and TLR3 (**B**) with mouse monoclonal antibodies in immunocomplexes after IP of these proteins with rabbit polyclonal antibodies from THP1 lysates, exposed to indicated doses of _γ_IR or treated with PMA or poly(I:C); 48 h post-irradiation. β-Actin in cell lysates, utilized for IP, was used as a reference. (**C** and **D**) Relative abundance of sense and antisense HERVK HML-2 *env* RNA in immunocomplexes after RNP-immunoprecipitation (RIP) of MDA-5 (**C**) and TLR3 (**D**) from THP1 lysates, shown on panels A and B. The fold change RNA count (ΔΔCt) was calculated in relation to β-actin reference RNA, detected in the lysates used for RIP. Error bars: ±SD of three independent replicates; * *p*<0.05, ** *p*<0.01, ^NS^ non-significant. (**E**) PLA assay visualizing association of HML-2 *env* RNA with MDA-5 receptor in THP1 in irradiated or control cells; 48h post-IR. Nuclei–DAPI staining, MDA-5/RNA complexes–Texas Red. Scale bars: 30 μm.

To quantitatively assess retroviral RNA binding to dsRNA receptors MDA-5 and TLR3, we employed an RNP-immunoprecipitation (RIP) method [100,101]. Western blot analysis of MDA5 and TLR3 in immune complexes pulled down from THP1 lysates, confirmed the increased level of both dsRNA receptors in the cells after exposure to _γ_IR (**Fig 6A** and **6B**). Treatment of the cells with PMA and especially poly(I:C) also raised levels of these receptors in the cytoplasm. The immune complexes isolated from irradiated cells were enriched in HML-2 *env* RNA (**Fig 6C** and **6D**). Similar levels of sense and antisense RNA in the same RIP complexes suggest binding of dsRNA duplexes. Consistent with the data in **Fig 3J**, relatively low levels of viral RNA were detected in TLR3 and MDA-5 immune complexes from PMA-treated cells, whereas poly(I:C) resulted in more viral RNA bound to dsRNA receptors, probably due to the feedback mechanism discussed below.

To validate Co-IP data and visualize HML-2 *env* RNA association with MDA-5, we utilized a proximity ligation assay, previously developed to analyze RNA-protein interactions (RNA-PLA) [102]. Use of a DNA oligonucleotide that hybridizes with both *env* RNA and another oligonucleotide coupled to anti-MDA-5 monoclonal antibody, allowed visualization of MDA-5 with viral RNA. Multiple, high intensity spots were detected in the cytoplasm of irradiated cells compared to non-irradiated ones (**Fig 6E**). These data suggest that HERV RNA, which is increased in the cytoplasm of monocytes and macrophages after therapeutically-relevant doses of _γ_IR, binds to dsRNA receptors and triggers downstream events resulting in IFN-α and inflammation-related cytokine expression, contributing to the biased macrophage polarization and secretory profile.

## Discussion

In the present study, we investigated how human monocytes and MDMs respond to radiation-induced stress, and revealed involvement of endogenous retroviruses in activation of signaling pathways that result in altered expression of inflammation-related genes and macrophage secretory factors (summarized in **Table 1**). While the exact mechanism of radiation-induced macrophage differentiation and polarization is not completely elucidated, secondary factors, such as released damage-associated molecular patterns (DAMPs), are considered to play a major role [12,103,104]. In our experiments, exposure of the monocytic THP1 cultures to _γ_IR doses led to death and cellular damage, which could, consequently, induce differentiation of nearby cells to a macrophage phenotype. Meanwhile, the data that radiation-mediated ROS production can be critical for macrophage differentiation [105,106], suggest that the direct radiation effect on ROS and nitric oxide production could also contribute to the differentiation of irradiated monocytes into macrophages.

**Table 1:** Expression and secretion of pro-inflammatory and anti-inflammatory macrophage markers in THP1 monocytes and primary monocyte-derived macrophages in response to gamma radiation.

Marker type	Marker gene/protein	SASP markers	Irradiated MDMs		Irradiated THP1		shRNA-Env	
			RNA	Protein	RNA	Protein	RNA	Protein
	IFNα		↑		↑		↓	
	IFNβ				↑		↓	
Proinflammatory	CD80		↑		↑	↑		
	CD86		↑		↑	↑	↓	
	HLA-DR		?			↑		
	NOS2	+	↑		↑			
	CCL2/MCP-1		↑	↑	↑	↑	↓	↓
	CCL3/MIP-1α		↑		↑	↑		↓
	CCL5	+	↓		↑		↓	
	CCL8/MCP-2		↑	?	↑		↓	↓
	CCL20/MIP-3α	+ +	↑		↑	↑	↓	↓
	CXCL1/GROα		↑		↑			
	CXCL8/IL-8	+		↑				↓
	CXCL10/IP-10			↑	↑			↓
	IL-1β		?		↑	↑	↓	↓
	IL-6	+	↑	↑	↑	↑	↓	↓
	IL-12				↑			
	IL-15		↑					
	TNF-α		↑	↑	↑	↑	?	↓
Anti-Inflammatory	CD11b		↑		↑	↑		
	CD163		↑	↑↓	↑	-		↓
	CD204		↑		-			
	CD206		↑		↑	↑		
	CCL22		↑		↑			
	IL-10		↑	↑↓	↑	↑	↓	↓
	TGF-β		↑		↑			
	ApoE	+			↑		↓	

+ indicated gene/protein is the marker of senescence-associated secretory phenotype.

↑ and ↓ increased and decreased gene expression or secretion of protein, respectively.

? gene expression changed in different directions in biological replicates of the same experiment.

Genes displayed stable HERV-related upregulation of expression are highlighted.

Macrophages are highly resistant to radiation [9,10] and are able to survive for days after exposure to doses reaching 10 Gy and massive DNA damage [4]. Our data showed that at least a fraction of the macrophage population reached cell cycle arrest and displayed expression of DNA damage-related senescence markers, such as p21^Waf1^ and GLB1. This correlated with a raised expression of SASP markers, most of which are inflammatory response factors [59]. These findings can explain conspicuous activation of inflammatory response after _γ_IR in both THP1 and MDMs. However, simultaneous increase of expression of the anti-inflammatory genes after radiation exposure, even in the terminally-differentiated MDMs that were initially polarized to anti-inflammatory phenotype by treatment with M-CSF, indicates involvement of additional molecular factors and pathways, related to IR, which in combination might contribute to development of a tumor-associated phenotype, at least in a fraction of the macrophage population.

Our data indicate that the complex response of macrophages to radiation is potentially related to dsRNA-induced signaling that triggers both IRF3-mediated IFN-I expression and NF-κB-dependent expression of the cytokine genes. Earlier studies have elucidated a critical role of IFN-I in increased IL-10 production and anti-inflammatory response of the human and mouse macrophages [30,107–109]. Although the upregulated IFNα response that we detected in human MDMs upon _γ_IR could be mediated by the STING/TBK1 pathway [74,76], increased expression of the cytoplasmic dsRNA receptors MDA-5 and TLR3 and the markers of activation of the downstream pathways suggests that dsRNA is also critical for the cellular response to radiation. Importantly, both receptors recognize long RNA ligands: TLR3 is shown to bind 90-to-540 kb dsRNAs [110], whereas MDA-5 forms multimolecular complexes with several kilobase-long duplex RNAs [25]. Another soluble cytosolic RNA sensor, RIG-I, can also bind dsRNA independently of the presence of 5′-triphosphate. However, the optimal length of these RNA duplexes is less than 300 nt [25]. Consistently, our findings suggest the presence of long dsRNA duplexes in the cytoplasm of irradiated macrophages.

Chiappinelli and colleagues found that DNA methyltransferase inhibitors used in cancer therapy triggered IFN-I response due to increased transcription of hypermethylated endogenous retroviruses, that resulted in accumulation of cytosolic dsRNA and induction of the dsRNA-sensing pathway [40]. Our RNA-seq and PCR analyses showed that a large number of HERVs, solo LTR’s, and retrotransposons were activated in THP1 and primary macrophages after radiation exposure. Many sequences contain deleterious mutations [89,111] and do not transcribe RNA molecules that are sufficiently long for efficient activation of the MDA-5 and TLR3 signaling pathways. We showed that at least two radiation-upregulated HERV subfamilies, HERVK HML-2 and HERV9NC, formed dsRNA duplexes. The evolutionarily youngest and most intact group of endogenous retroviruses, HML-2 displayed elevated level of the long antisense transcripts upon _γ_IR, suggesting that various HERV loci transcribing the few kilobase-size RNAs in both directions can serve as a source of dsRNA molecules in the irradiated cells. High levels of antisense HERV transcripts have been earlier reported [41,85]. Antisense HERV transcripts can be generated if proviral sequences were integrated in the opposite direction within introns of host genes or via read-through transcription downstream of a transcribed host gene or repetitive element [85]. Predominance of the shorter *env*-encoded HML-2 RNAs can be a result of host promoter-driven transcription of the minus-strands of the proviruses with deleted *gag* and *pro-pol* regions. Alternatively, the minus-strand 3’LTR could drive transcription of the antisense sequence, as has been shown for other retroviruses [91,112–118].

Association of HML-2 *env* RNA with the TLR3 receptor suggests that at least some viral dsRNA becomes localized in the endosomal compartment. We previously showed that the HIV-1 transactivating response (TAR) element RNA, rich in double stranded stem-loop structures, is secreted from infected cells in extracellular vesicles called exosomes and activates TLR3 signaling in uninfected macrophages [21]. Later data showed that longer HIV-1 transcripts were also abundant in exosomes released from HIV-1 infected monocytes [119]. The fact that long ncRNAs are transported by exosomes and can affect the recipient cells [120] suggests that HERV-encoded dsRNA could not only activate dsRNA receptors in the producing cells, but could also be incorporated into extracellular vesicles and activate TLR3 in the recipient cells, including cells in non-irradiated tissues.

Partial knockdown of *env* from HML-2 resulted in reduced IFNα and cytokine expression after γIR, indicating that this RNA triggers the dsRNA-induced IFN-I and cytokine response. Surprisingly, although we used HML-2 *env*-specific shRNAs, the RNA level of some other HERVs was also reduced. This can explain the remarkable reduction of interferon and cytokine expression in response to HML-2 shRNA. However, this phenomenon raises questions about the mechanism of observed inhibition. The published study of HERV-K expression in ALS brain tissue revealed functional activity of interferon-stimulated response elements (ISREs) in the viral promoter: the authors demonstrated synergistic upregulation of HERV-K transcription by IRF1 and NF-κB isoforms [121]. Recently, a publication by Cañadas and colleagues identified a novel functional group of ERVs called Stimulated 3 prime antisense retroviral coding sequences (SPARCs) [42]. These are proviruses that are inversely oriented in 3′ untranslated regions of specific interferon-stimulated genes, silenced by an H3 histone methyltransferase EZH2, but can be activated in response to IFN_γ_ stimulation, due to the presence of STAT1-binding sequences in the promoters of the host genes and 5′LTR in the ERV minus-strand [42]. This results in the generation of dsRNA that in turn can activate dsRNA sensors and subsequent IFN induction. Accordantly, these data suggest a positive feedback signal amplification that represents a potential mechanism of enhancement of pathologic innate immune signaling in irradiated macrophages. Conversely, even if a fraction of HERV transcripts is reduced, it can result in further reduction of the transcription of ERV elements whose promoter regions contain IRF and NF-κB binding sites and, therefore, reduced dsRNA signaling and expression of related genes. While most of the HERV clades we found upregulated in monocytes and MDMs do not belong to the SPARC group, their LTR sequences are polymorphic and contain multiple transcription factor-binding sites, including IRF1, IRF5 and STAT3 [121,122]. Thus, their activation, at least in part, can also be dependent on the positive feedback loop, triggered by IFN-I via IFNAR1 and probably some pro-inflammatory cytokines.

In conclusion, transcription of both the positive and negative strands of various HERV subfamilies activated by gamma radiation in heterogeneous population of monocytes and monocyte-derived macrophages leads to formation of long dsRNA molecules that bind to cytoplasmic receptors MDA-5 and TLR3, which in turn induce signaling pathways resulting in enhanced expression of type I interferons and a biased expression of macrophage secretory markers, cytokines and chemokines, involved in both pro-inflammatory and anti-inflammatory response. Thus, elevated expression of endogenous retroviruses may determine polarization of the macrophages toward an altered pro-inflammatory phenotype, while maintaining regenerative or oncogenic activity in the context of radiation therapy.

## Materials and methods

### Cells

The cells and reagents utilized in this study are listed in **Table 2**. The acute monocytic leukemia cell line THP1 (from S. Tsuchiya; provided by the NIH AIDS Research & Reference Reagent Program) and the human kidney fibroblasts 293T/17 (ATCC, Manassas, VA) were maintained at 37°C and 5% CO_2_ in RPMI-1640 culture media (THP1) or DMEM (293T/17) supplemented with 10% FBS, penicillin/streptomycin, and L-Glutamine. The reporter cell lines THP1-Dual and THP1-Dual KO-IFNAR2, both from InvivoGen, were maintained in RPMI-1640 culture media with additional 100 μg/ml Normocin according to the manufacturer’s protocol. Fractions of human blood samples enriched with the white blood cells were obtained from The NIH Blood Bank. MDMs were prepared from PBMCs of healthy individuals using adherence to plastic, and differentiated in the presence of M-CSF as described earlier [123]. The cells were allowed to differentiate for 7 days in Dutch modified RPMI-1640 culture medium (Gibco) supplemented with 10% human serum (Corning), penicillin/streptomycin, sodium pyruvate and L-Glutamine in the presence of 10 nM M-CSF (PeproTech). Half of the medium was replaced every third day and after day 8, cells were cultivated for 14–21 days without additional growth factors. Where indicated, PMA was added at a 20 nM final concentration and poly(I:C) was added at a 2 μg/ml final concentration.

**Table 2:** Key resources.

REAGENT or RESOURCE		SOURCE	IDENTIFIER
**Antibodies**			
β-Actin (clone AC-15)		Millipore Sigma	A5441
GAPDH (clone 0411)		Santa Cruz Biotechnology	sc-47724
CD11b (clone D12), APC		BD Biosciences	340936
CD206 (MMR), (clone 15–2), PE/Cy7		BioLegend	321124
CD86 (clone BU63), APC		BioLegend	374208
CD80 (B7-1), (clone 2D10.4), FITC, eBioscience		Thermo Fisher Scientific	11-0809-42
IRF-3 (clone D614C)		Cell Signaling Technology	1190
IKKε (clone D20G4)		Cell Signaling Technology	8348
Integrin β1 (clone 4B7R)		Santa Cruz Biotechnology	Sc-9970
HLA-DR (clone LN3)–PE-Cyanine5, eBioscience		Thermo Fisher Scientific	15-9956-42
MAVS (polyclonal)		Cell Signaling Technology	3993
MDA-5 (clone D74E4)		Cell Signaling Technology	5321
MDA-5 (goat polyclonal)		Abcam	Ab4544
IFIH1/MDA-5 (rabbit polyclonal)		Proteintech	21775-1-AP
p21 Waf1/Cip1 (clone 12D1)		Cell Signaling Technology	2947
Phospho- IRF3 (Ser396) (clone4D4G)		Cell Signaling Technology	4947
Phospho- IKKε (Ser172) (clone D1B7)		Cell Signaling Technology	8348
Phospho-Stat1 (Tyr701) (clone 58D6)		Cell Signaling Technology	9167
Phospho-Stat1 (Ser727) (clone D3B7)		Cell Signaling Technology	8826
RIG-1 (clone D33H10)		Cell Signaling Technology	4200
Stat1 (clone D1K9Y)		Cell Signaling Technology	14994
TBK1/NAK (D1B4)		Cell Signaling Technology	3504
Toll-like Receptor 3 (clone 40C1285)		Abcam	Ab13915
Toll-like Receptor 3 (clone D10F10)		Cell Signaling Technology	6961
Toll-like Receptor 8 (polyclonal)		Abcam	Ab180610
Toll-like Receptor 7 (clone D7)		Cell Signaling Technology	5632
Anti dsRNA (clone rJ2)		Millipore Sigma	MABE 1134
Light Diagnostics Pan-enterovirus reagent (clone 9D5)		Millipore Sigma	3361
**Cloning vectors, Bacterial and Virus Strains**			
**NEB Stable Competent** *E*.*coli*		New England Biolabs	C3040I
pLKO.1 puro (from Bob Weinberg)		Addgene	8453
psPAX2 (from Didier Trono)		Addgene	12260
pMD2.G (from Didier Trono)		Addgene	12259
pCR4-TOPO vector		ThermoFisher	K457502
**Chemicals, commercial siRNA, Dyes, Peptides, and Recombinant Proteins**			
Phorbol-12-myristate-13-acetate (PMA)		Millipore Sigma	524400
Polyinosinic acid (Poly (I:C))		Millipore Sigma	528906
DSG (disuccinimidyl glutarate)		Thermo Fisher Scientific	20593
IFN alpha- IFNAR-IN-1 hydrochloride		MedChemExpress	HY-12836A
Poly-L-lysine solution		Millipore Sigma	P4707
Recombinant Human M-CSF		PeproTech	300–25
TRIzol Reagent		Thermo Fisher Scientific	15596026
Phosphate-buffered 4% paraformaldehyde		FDNeuroTechnologies	PF 101
FcR Blocking Reagent		Miltenyi Biotec	130-059-901
DRAQ5 Fluorescent Probe		Thermo Fisher Scientific	62252
CellBrite Green Cytoplasmic Membrane Dye		Biotium	30021
Rhodamine Phalloidin		Thermo Fisher Scientific	R415
MitoSox Red mitochondrial superoxide indicator		Thermo Fisher Scientific	M36008
DNase I, RNase-free		Thermo Fisher Scientific	EN0521
RNase Recombinant Ribonuclease Inhibitor		Thermo Fisher Scientific	N8080119
Halt Protease Inhibitor Cocktail		Thermo Fisher Scientific	78429
Silencer Select Pre-Designed siRNA, Gene Symbols: IFNA4, IFNA17,IFNA10, IFNA7		Thermo Fisher Scientific	s230838
Silencer Select Pre-Designed siRNA, Gene Symbol: IFNA2		Thermo Fisher Scientific	s7158
Silencer Select Pre-Designed siRNA, Gene Symbol: IFNB1		Thermo Fisher Scientific	s7187
Silencer Select Pre-Designed siRNA, Gene Symbol: IFIH1		Thermo Fisher Scientific	s34500
Silencer Select Negative Control No. 1 siRNA		Thermo Fisher Scientific	4390843
DOTAP Liposomal Transfection Reagent		Millipore Sigma	11202375001
**Critical Commercial Assays**			
Duolink In Situ Red Starter Kit Mouse/Rabbit		Millipore Sigma	DUO92101
Duolink In Situ PLA Probe Anti-Rabbit Plus		Millipore Sigma	DUO92002
Coelenterazine-utilizing luciferase detection medium		InvivoGen	rep-glc2
Bio-Plex Pro Human Cytokine Screening Panel		Bio-Rad	N/A
Bio-Plex Pro Human Chemokine Assays		Bio-Rad	N/A
RT^2^ Profiler PCR Array Human Type I Interferon Response		Qiagen	330231
Pierce BCA Protein Assay Kit		Thermo Fisher Scientific	23225
SsoAdvanced Universal SYBR Green Supermix		Bio-Rad	172–5272
**Deposited Data**			
Raw sequence data (FASTQ files) of transcriptome profiling and enumerated counts for each gene and transposable element for each sample are available at the NCBI Gene Expression Omnibus		https://www.ncbi.nlm.nih.gov/geo/query/acc.cgi?acc=GSE145577	GSE145577
**Experimental Models: Cell Lines, primary cells**			
THP1 Human monocytes, acute monocytic leukemia		ATCC	ATCC-TIB-202
THP1 -dual NF-kB-SEAP IRF_Luc reporter Monocytes		InvivoGen	thpd-nfis
THP1-dual-ko-IfnaR2 cells		InvivoGen	thpd-koifnar2
PBMC, Homo sapiens		NIH Blood Bank	
**Oligonucleotides**			
**Name**	**Sequence**		
*shRNAs*			
shRNA HERV-K HML-2 env1	CCTGAACATCCAGAATTAT	[124]	
shRNA HERV-K HML-2 env1-Control	GAATTCTTAACGACTACCA	[124]	
shRNA HERV-K HML-2 env1070	CCTCGAGGTCAATTCTACCACAATT	BLOCK-iT RNAi Designer (https://rnaidesigner.thermofisher.com/rnaiexpress/)	
shRNA HERV-K HML-2 env1070-Control	CCTAGGTCAATTCTACCACACGATT		
*HERV-specific primers*			
ERVF-R	TAAAGAGGGCATGGAGTAATTGA	[42]	
ERVL-F	ATATCCTGCCTGGATGGGGT		
ERVL-R	GAGCTTCTTAGTCCTCCTGTGT		
MLT1B-F	TGCCTGTCTCCAAACACAGT		
MLT1B-R	TACGGGCTGAGCTTGAGTTG		
MLT1C49-F	TATTGCCGTACTGTGGGCTG		
MLT1C49-R	TGGAACAGAGCCCTTCCTTG		
MLT1C627-F	TGTGTCCTCCCCCTTCTCTT		
MLT1C627-R	GCCTGTGGATGTGCCCTTAT		
MER4D-F	CCCTAAAGAGGCAGGACACC		
MER4D-R	TCAAGCAATCGTCAACCAGA		
Mer57B1-F	CCTCCTGAGCCAGAGTAGGT		
Mer57B1-R	ACCAGTCTGGCTGTTTCTGT		
MTL2B4-F	GGAGAAGCTGATGGTGCAGA		
MTL2B4-R	ACCAACCTTCCCAAGCAAGA		
MLTA10-F	TCTCACAATCCTGGAGGCTG		
MLTA10-R	GACCAAGAAGCAAGCCCTCA		
MLT1A-F	TGGGCTCTTTGGGTGATAGT		
MLT1A-R	TTGCAGATGGCTACCTTCCT		
MLT1J-F	GCTGTTGCACACATGCTCTT		
MLT1J-R	GATGTGGAAGTCTGGGAAGC		
THE1D-F	CACCCTGCTTCTCCTGCT		
THE1D-R	AATGCCTGAGACTGGGTGAT		
MLT1K-F	CAGATGAAGCCCTCTCTCAGA		
MLT1K-R	CCTTCCTCTCTCCTGGTGTC		
PABL_B-F	GAAGGCACATAACCCCAACC		
PABL_B-R	GGGTCCAGCTGTGTTTTCTG		
MLT1A0-F	ATGAATGGGATTGGTGGGCT		
MLT1A0-R	TTCTAGAGCCTGGGAAGTCC		
MSTA-F	TAACTGGGTCATGAGGGTGG		
MSTA-R	CATTTGCTCGGATTCTGGGG		
LTR79-F	AACTCTGGGCTTCCGTTTCC		
LTR79-R	AAAGCATGCCTCTTTCCTGC		
MER92B-F	GTTAAGCTTCCCTCCTCCCC		
MER92B-R	AGTGAAAAGGCTCAGACCGA		
MLT1I-F	TTCCCTCTGCCCTGATCTAA		
MLT1I-R	GTCAGTGGCTTACAACAACGT		
LTR26-F	CTCCAAGGAATTGACTCAGCA		
LTR26-R	TCTACCTCCCTGCTGAGTCT		
Env-E4-1F	CACGGTGGAAAGACCGTAGT	[40]	
Env-E4-1R	CAGTCAGCTTCTGGGTGTGA		
Sync-3-F	TGCACCTACATTCCCAACAA		
Sync-3-R	GCGATTGCGAGAGAGGTAAC		
Env-V1-F	GTCAGCAAAAGGAGGAGCGT		
Env-V1-R	TGGCTGGGCCTTGATAAGTT		
Gag-W5-F	TAACTGCAGCCCGAGAGTTT		
Gag-W5-R	GGCACCAATCTCCATGTTCT		
Pol-ERVF-F	ATCTCCTCACTGCCGAGAAA		
Pol-ERVF-R	TCTCGATCTCCTGACCTCGT		
Env-W2-F	GTTGTCCTGGAGGACTTGGA		
Env-W2-R	GCCGAGTGAGGGTGGTATTA		
HERV-Fc1-F	TGCAGAAGACAAGGCAATG		
HERV-Fc1-R	AGTGTTCCCTTGGACAGGTG		
Env-E4-1	CACGGTGGAAAGACCGTAGT		
HERV-Kenv-F	CTGAGGCAATTGCAGGAGTT	[88]	
HERV-Kenv-R	GCTGTCTCTTCGGAGCTGTT		
HERV-Kpol-F	TCACATGGAAACAGGCAAAA		
HERV-Kpol-R	AGGTACATGCGTGACATCCA		
HERV-Kgag-F	AGCAGGTCAGGTGCCTGTAACATT		
HERV-Kgag-R	TGGTGCCGTAGGATTAAGTCTCCT		
HERV-K_LTR-F	AGGGAAAAACCGCCTTAGGG		
HERV-K_LTR-R	AGCAGACAAACATGTGAACAAAGG		
HML-2_1778_for1	CCCCCAGAAAGTCAGTATGGA	[127]	
HML-2_1778_for2	TCTCCAGAGGTTCAGTATGGA		
HML-2_1778_for3	CCCCCAGAAAATCAGTATGGA		
HML-2_1778_for4	TCTCCAGAGGTGCAGTATAGA		
HML-2_2396_rev1	TTTCCCAGGCTCTAAGGCAG		
HML-2_2396_rev2	TTCCCAGGCCCTGAGGCAA		
HML-2_2396_rev3	TTTCCTAGGCTCTAAGGCAG		
K-Int-d120-F	GATGCTATTGTCAGGCCTGC		
K-Int-d120-R	GTGGAAAGTGTTGCCTCAGG		
K-Int-d121-F	TACCGGATATTGCCATGCCT		
K-Int-d121-R	AGTATGGATCTCAGGCGGTG		
K9-Int-d366-F	CGCAAAAGGTTCCAACAGGA		
K9-Int-d366-R	CATACGGCACAATCAGGAGC		
K9-Int-d367-F	TCCAGCCCAAAATCAACAGC		
K9-Int-d367-R	GTTGCTCATGACAGTTGGGG		
MER9a-d169-F	AACAATCTCTGCAGCACTGG		
MER9a-d169-R	GGCACAGATCGCTCATGTTA		
K3-d113-F	GCCACAACTCCATTTCAGGG		
K3-d113-R	AAAATCAACAACTCCCGGG		
HERVP71a-Pol-F	CATAGTTCGCATAAATGGTCATC		
HERVP71a-Pol-R	CTGGGGCCTTAATACTACCTTAG		
HERVP71a-Gag-F	ACCCAGTGAAAAGCCCTGGGAC		
HERVP71a-Gag-R	CAGTTGGCTTGGCTCCCCTGTAG		
PRIMA41-Pol-F	CAAAGGTTAAGGAAGTTTCTCAG		
PRIMA41-Pol-R	GAGGTGAGCTTAAGGCTGCAG		
HERV9NC-Pol-F	AGGAGAAAGCTCCAAAAGCAAGC		
HERV9NC-Pol-R	GGTGATTGGCCTGCTCCATTTTC		
HERV9NC-Env-F	TCTCAAGTCCAGGCACTCTGGTC		
HERV9NC-Env-R	TTCCTTGCTGAGGGCCCTGGTC		
HERVK3-Env-F	GCTATGTCAACATTTTGTAGGACAG		
HERVK3-Env-R	GCTATTTTGAGATTCCATTTAGTCAAAGC		
HERV4-Pol-F	GACTCAACCCCTATAGCACAAG		
HERV4-Pol-R	TGAGGGCTGTAGCTTTGGCTTC		
HERV4-Pol2-F	ACATTCAGCATGGAGGCCTCAATC		
HERV4-Pol2-R	GAGGGCTATGGGCAGTAAGCTG		
*Primers for reverse transcription (antisense RNA)*			
K-anti-env6829-R	CTGATCTAGAGGTACCGGATCGAAACCTTGCCCCAAGGAAATTC		
K-anti-env6433ss-R	CTGATCTAGAGGTACCGGATCACATTTGAAGTTCTACAATGAACCCATC		
K-anti-env7989-R	CTGATCTAGAGGTACCGGATCCTGAGGCAATTGCAGGAGTT		
K-anti-Gag-1259-R	CTGATCTAGAGGTACCGGATCCCAGAACAAGGAACTTCAGATCTAAAA		
K-anti-Gag-2908-R	CTGATCTAGAGGTACCGGATCGATAAAAATGGGCAACCATTGTCG		
K-anti-Pol-4545-R	CTGATCTAGAGGTACCGGATCCTGGACTGGCAATAGCATCCGATA		
*Primers for anti-sense RNA*			
K-TAG-R	CTGATCTAGAGGTACCGGATC		
K-anti-env7023-F	GGACTCACTTGTGCACTTGG		
K-anti-env6669-F	CATCCTGGTGCTCTCCCTAG		
K-anti-Gag-1432-F	GGCATCAGAAACTGAAATGCTATCTTC		
K-anti-Gag-3097-F	GTACATAAATCTACTGCTGCACTGC		
K-anti-Pol-4742-F	TGGCATAAGTAGGAATGCCTAGAG		
*Primers for cellular genes (human)*			
hGAPDH-F	TGCACCACCAACTGCTTAGC		
hGAPDH-R	GGCATGGACTGTGGTCATGAG		
B-actinF	GTGGGGCGCCCCAGGCACCA		
B-actinR	CTCCTTAATGTCACGCACGATTTC		
G6PDH-F	ATCGACCACTACCTGGGCAA		
G6PDH-R	TTCTGCATCACGTCCCGGA		
hIL1-beta-F	AAGCTGATGGCCCTAAACAG		
hIL1-beta-R	AGGTGCATCGTGCACATAAG		
hIL6-F	CCAGCTATGAACTCCTTCTC		
hIL6-R	GCTTGTTCCTCACATCTCTC’		
hTNF-a_F	ATGAGCACTGAAAGCATGATCC		
hTNF-a_R	GAGGGCTGATTAGAGAGAGGTC		
hIL10-F	TCAAGGCGCATGTGAACTCC		
hIL10-R	GATGTCAAACTCACTCATGGCT		
hTGF-beta1_F	CCCAGCATCTGCAAAGCTC		
hTGF-beta1_R	GTCAATGTACAGCTGCCGCA		
hIL18-F	TCTTCATTGACCAAGGAAATCGG		
hIL18-R	TCCGGGGTGCATTATCTCTAC		
hCD80-F	GGCCCGAGTACAAGAACCG		
hCD80-R	TCGTATGTGCCCTCGTCAGAT		
hCD86-F	CTGCTCATCTATACACGGTTACC		
hCD86-R	GGAAACGTCGTACAGTTCTGTG		
hIFNa-F	GACTCCATCTTGGCTGTGA		
hIFNa-R	TGATTTCTGCTCTGACAACCT		
hIFNb1-F	GCTTGGATTCCTACAAAGAAGCA		
hIFNb1-R	ATAGATGGTCAATGCGGCGTC		
hIL8-F	TTTTGCCAAGGAGTGCTAAAGA		
hIL8-R	AACCCTCTGCACCCAGTTTTC		
hIL12a-F	CCTTGCACTTCTGAAGAGATTGA		
hIL12a-R	ACAGGGCCATCATAAAAGAGGT		
hCCL2-F	GAGAGGCTGAGACTAACCCAGA		
hCCL2-R	ATCACAGCTTCTTTGGGACACT		
hCCL2b-F	CCCCAGTCACCTGCTGTTAT		
hCCL2b-R	TGGAATCCTGAACCCACTTC		
hCXCL10-F	GTGGCATTCAAGGAGTACCTC		
hCXCL10-R	TGATGGCCTTCGATTCTGGATT		
hCCL5-F	CCAGCAGTCGTCTTTGTCAC’		
hCCL5-R	CTCTGGGTTGGCACACACTT		
hCD11b-F	CAGCCTTTGACCTTATGTCATGG		
hCD11b-R	CCTGTGCTGTAGTCGCACT		
hCD206-F	GGGAAAGGTTACCCTGGTGG		
hCD206-R	GTCAAGGAAGGGTCGGATCG		
hCD163-F	TTTGTCAACTTGAGTCCCTTCAC		
hCD163-R	TCCCGCTACACTTGTTTTCAC		
hCD204-F	CCTGTGCATTGATGAGAGTGC		
hCD204-R	TGCTCCATACTTCTTTCGTCCT’		
hCCL20-F	TGCTGTACCAAGAGTTTGCTC		
hCCL20-R	CGCACACAGACAACTTTTTCTTT		
hCCL3-F	AGTTCTCTGCATCACTTGCTG		
hCCL3-R	CGGCTTCGCTTGGTTAGGAA		
hCCL8-F	TGGAGAGCTACACAAGAATCACC		
hCCL8-R	TGGTCCAGATGCTTCATGGAA		
hCXCL1-F	GCGCCCAAACCGAAGTCATA		
hCXCL1-R	ATGGGGGATGCAGGATTGAG		
hGLB1-F	TATACTGGCTGGCTAGATCACTG		
hGLB1-R	GGCAAAATTGGTCCCACCTATAA		
hCDKN1A-F	CGATGGAACTTCGACTTTGTCA		
hCDKN1A-R	GCACAAGGGTACAAGACAGTG		
huAPOE-F	GTTGCTGGTCACATTCCTGG		
huAPOE-R	GCAGGTAATCCCAAAAGCGAC		
**Recombinant DNA**			
pLKO.1-HERVK_LTR-puro			
pLKO.1-HERVK_Env1-puro			
pLKO.1-HERVK_Env1070-puro			
**Software and Algorithms**			
SnapGene		www.snapgene.com	
GeneAssist Custom siRNA Builder		https://www.thermofisher.com/order/custom-genomic-products/tools/sirna/	
FlowJo		www.flowjo.com	
INSPIRE		www.amnis.com	
CFX Manager Software v3.1		https://www.bio-rad.com/en-us/product/previous-qpcr-software-releases?ID=OO2BB34VY&source_wt=cfx-manager-software_surl	
Bio-Plex Manager Software 6.2		https://www.bio-rad.com/en-us/product/bio-plex-manager-software-standard-edition?ID=5846e84e-03a7-4599-a8ae-7ba5dd2c7684	
FISH probe design tool		http://prober.cshl.edu/	
ImageJ		https://imagej.nih.gov/	
Bcl2fastq2 conversion software 2.20		https://support.illumina.com/downloads/bcl2fastq-conversion-software-v2-20.html	
FastQC		http://www.bioinformatics.babraham.ac.uk/projects/fastqc/	
Trimmomatic		http://www.usadellab.org/cms/?page=trimmomatic	
STAR		https://github.com/alexdobin/STAR	
Samtools		http://samtools.sourceforge.net/	
Ensembl		ftp://ftp.ensembl.org/pub/release-99/gtf/homo_sapiens/Homo_sapiens.GRCh38.99.gtf.gz	
TEtranscripts		http://labshare.cshl.edu/shares/mhammelllab/www-data/TEtranscripts/TE_GTF/GRCh38_rmsk_TE.gtf.gz	
TEcount		https://pypi.org/project/TEtranscripts/	
R		https://cran.r-project.org/	
*IDEAS (*Image Data Exploration and Analysis Software) version 6.2		www.amnis.com	
OriginPro 2019		www.originlab.com	
ClustalW (CLCbio Genomics Workbench, v11)		www.qiagen.com	
JalView		https://www.jalview.org/	
**Other**			
Fixation buffer		BioLegend	420801
RIPA buffer		Thermo Fisher Scientific	89900

### Plasmids, shRNA expressing lentiviral vectors and transduction

The 3^rd^ generation puromycin resistant lentiviral backbone pLKO.1 puro (Addgene) was used for cloning and expression of HML-2 *env* shRNA sequences. HML-2 *env*-targeting shRNAs and matching control shRNAs were designed for conservative regions in the 5’LTR and *env* gene of the HERV-K10 sequence (GenBank No. M14123.1) using the Invitrogen RNAi Designer tool. Two shRNA sequences with the highest predicted score and shRNA-Env from [124] were cloned under control of human U6 promoter into the pLKO.1 puro vector using AgeI and EcoRI restriction enzyme sites following Addgene protocol for pLKO.1 - TRC Cloning Vector. Lentiviruses were produced by cotransfection with MISSION lentiviral packaging mix (Sigma) into 293T/17 cells using Lipofectamine 3000 (Invitrogen). Lentivirus-containing culture media was collected from transfected 293T/17 cells at 48- and 72-hours post-transfection. The samples were centrifuged at 2,500 x *g* for 10 min and filtered (0.22 μm pore). Viral titers were normalized to 0.01 pg of p24^CA^ per cell, using a p24 ELISA kit (PerkinElmer). The virus suspensions were then either stored at −80°C or used immediately to infect THP1 cells in combination with 8 μg/ml polybrene by spinoculation at 1,000 x *g* and 25°C for 2 h, as described earlier [125]. Virus-transduced THP1 cells were cultured after selection with puromycin (0.5 μg/ml; Gibco). Lentiviral transduction of primary MDMs was performed in 24-well plates by overnight incubation of viral suspensions in Dutch-modified RPMI-1640 culture medium with Polybrene (Sigma) in a final concentration of 8 μg/ml.

### Transfections

The Lipofectamine RNAiMAX reagent protocol (Thermo Fisher) was used to transfect cells with siRNA. Briefly, 0.75 × 10^6^ THP1 cells (50%-confluent) were transfected with the complexes containing 10 nM siRNA and 6 μl Lipofectamine RNAiMAX in 150 μl of Opti-MEM medium. The cells were incubated with siRNA–lipid complexes for 4 h, after which the mixture was aspirated and replaced with 1 ml of complete culture medium. Poly(I:C) (1 μg) was transfected into the cells with 6 μl Lipofectamine 3000 (Thermo Fisher) in 150 μl of Opti-MEM medium, according to the manufacturer protocol. Subsequent steps were the same as described above. For the transfections with the DOTAP Liposomal Transfection Reagent (Roche), 1 μg poly(I:C) was mixed with 8 μl of HBS buffer (20 mM HEPES, cell culture grade, 150 mM NaCl, pH 7.4) and 6 μl of DOTAP in 12 μl of HBS buffer. The cells were mixed with transfection complex in 0.3 ml of Opti-MEM medium, incubated for 4 h at 37°C and then cultured in the regular media for 48 h.

### Gamma irradiation

The irradiation experiments were performed in a ^137^Cs irradiator (JL Shepherd Associates, San Fernando, CA, USA) at ambient temperature (22–25°C) and atmosphere. The dose rate during all experiments was approximately 34 Gy/h; however, the exact irradiation time was calculated on the day of each experiment.

### Flow cytometry

For the surface staining, THP1 cells were washed with HBSS buffer (Gibco) supplemented with 2% FBS and blocked with human FcR blocking reagent (Miltenyi Biotec) according to manufacturer’s instruction. Then, the cells were incubated for 30 min on ice with optimal concentration of one of the following anti-human antibodies in HBSS+2% FBS buffer: HLA-DRPECy5 (LN3), CD163-PE(eBioGHI/61), CD80-FITC (2D10.4) (Thermo Fisher Scientific), CD207-PECy7 (15–2) (BioLegend) or CD11b-APC (D12) (BD Biosciences). For MitoSox Red live-cell staining, the DPBS-washed THP1 cells were stained with 5μM MitoSox Red (Thermo Fisher Scientific) in DPBS for 15 min at 37°C and 5% CO_2_. To perform staining, the cell pellets were resuspended in 1 μM DAPI solution in HBSS+2% FBS after triple wash, filtered through nylon mesh and analyzed using LSRII Flow cytometer (BD Biosciences). Data analysis was performed with the BD FACSDiva or FlowJo software. Doublets (on FSC-H vs FSC-A dot plot), cell debris (gated out on side vs forward scatter dot plot) and dead (DAPI^high^) cells were excluded from analysis. Fluorescent-activated cell sorting was performed on FACSAria Fusion Cell Sorter (100μm nozzle configuration). Post-sort cell purity exceeded 99%. Histogram data represent percent of positively stained single viable cells over background.

### Imaging flow cytometry

For the imaging flow cytometry, washed as described above THP1 cells were incubated with 30 μM DNA-intercalating membrane permeable dye DRAQ5 (Thermo Fisher Scientific) in DPBS solution for 20 min at room temperature and then analyzed using MARKII ImageStream Imaging Cytometer. Data were acquired using INSPIRE Software at 60x magnification. The IDEAS software version 6.2 was used for data analysis. The number of granules was determined by utilizing the spot counting feature in IDEAS. Focused, single cells containing DNA dye were selected for the spot counting.

### RNA isolation, reverse transcription and quantitative PCR (RT-qPCR) analysis

Total RNA was extracted from cells or precipitated immune complexes using Trizol Reagent (Thermo Fisher Scientific) via the addition of a minimum of nine volumes of the reagent to the washed cell pellets or IP complexes, resuspended in DPBS or to the adherent cells washed directly in the wells of tissue culture plates. Total RNA was then isolated according to manufacturer protocol, precipitated with isopropanol and 20 μg of glycogen (Roche) and washed with 75% ethanol. The RNA pellets were air dried, reconstituted in water and then treated with DNase I RNase-free (Invitrogen), 1U per 1 μg DNA, for 20 minutes at 37°C in the presence of 5mM MgCl_2_ and RNAse Inhibitor. Total RNA was re-purified using Trizol reagent or RNeasy Mini Kit (Qiagen) following the manufacturer’s protocol. After resuspending in RNase-free water, a 500-to-2,000 ng of total RNA was used to generate cDNA with the High Capacity cDNA Reverse Transcription Kit (Thermo Fisher Scientific), 10U of RNase Inhibitor (Applied Biosystems) and either oligo-dT-primer or one of the reverse transcription primers indicated in **Table 2**, following the manufacturer protocol. The RT reaction was performed with and without reverse transcriptase in the reaction mixture to ensure genomic DNA removal for qPCR analysis. Quantitative real-time PCR was performed with 2 μl of minimum 2-fold diluted RT reaction mixtures, the SsoAdvanced Universal SYBR Green Supermix (Bio-Rad) and the primers specific for particular genes **Table 2**). The optimal PCR program was as follows: 95°C for 3 min; then 95°C for 10 sec, 60°C for 40 sec and ran 41 cycle for both the tested and reference genes. Real-time PCR reactions were carried out at least in triplicate. Relative gene expression was determined by the _ΔΔ_Ct ratio method using BioRad CFX Manager 3.1 software. The fold gene expression in all experiments was calculated in relation to β-actin as a reference gene. The PCR efficiencies for the primer sets were estimated to be 100 ± 10% by titration analysis [126].

### Pathway-focused gene expression analysis using PCR array

RNA was isolated from the cells as described above, treated with DNAse I, re-purified using RNeasy Mini Kit (Qiagen) and subjected to reverse transcription reaction with random hexamer primers as described above. Real-time PCR was performed in 96-well plates from RT^2^ Profiler PCR Array Human Type I Interferon response kit (Qiagen) with 1 μl of cDNA equivalent to 50 ng RNA for each reaction and SsoAdvanced Universal SYBR Green Supermix (Bio-Rad) according to the manufacturer protocol (Qiagen). Relative gene expression against the β-actin housekeeping gene was determined by the _ΔΔ_Ct ratio method and presented on volcano plots.

### Total RNA transcriptome profiling by next-generation sequencing

Total RNA was extracted from non-irradiated or 5 Gy irradiated THP1 cells (three biological replicates of each sample) or primary human MDMs (from four healthy donors) as described above. After DNase I treatment, RNA was re-purified using RNeasy Plus Mini Kit (Qiagen) following manufacturer instruction. The total RNA integrity was assessed using automated capillary electrophoresis with a Fragment Analyzer (Agilent). For all samples with an RQI > 8.0, a total of >75 ng RNA was used as input for library preparation using the TruSeq Stranded Total RNA Library Preparation Kit (Illumina, San Diego, CA). The sequencing libraries were quantified by quantitative PCR using a KAPA Library Quantification Kit for NGS (Kapa Biosystems) on a Roche Light Cycler 480 Instrument II and the size distribution was assessed with a Fragment Analyzer. The sequencing libraries were pooled and sequenced on a NextSeq 500 Desktop Sequencer (Illumina) using a NextSeq 500 High Output Kit v2 with paired-end reads of 75 bp in length. The raw sequencing data were demuxed using bcl2fastq2 conversion software 2.20 (Illumina).

### Sequence mapping and feature enumeration

Raw sequence reads were quality inspected via FastQC (http://www.bioinformatics.babraham.ac.uk/projects/fastqc/) then adaptor clipped (TruSeq3-PE-2.fa:2:30:10) and quality trimmed (HEADCROP:13 SLIDINGWINDOW:4:20 MINLEN:15) using Trimmomatic (http://www.usadellab.org/cms/?page=trimmomatic). Reference mapping was performed using surviving read pairs against GRCh38.98 (—sjdbOverhang 74 -winAnchorMultimapNmax 100—outFilterMultimapNmax 100—outSAMtype BAM SortedByCoordinate) using STAR (https://github.com/alexdobin/STAR). After, mapping files were indexed using samtools (http://samtools.sourceforge.net/) and the number of counts per known annotated gene (ftp://ftp.ensembl.org/pub/release-99/gtf/homo_sapiens/Homo_sapiens.GRCh38.99.gtf.gz) and annotated transposable element (http://labshare.cshl.edu/shares/mhammelllab/www-data/TEtranscripts/TE_GTF/GRCh38_rmsk_TE.gtf.gz) were enumerated (—sortByPos—format BAM—stranded reverse—mode multi) using the TEcount tool (https://pypi.org/project/TEtranscripts/). All commercial software tools used in this study are indicated in **Table 2**.

### Transcriptome analysis

Enumerated counts for known annotated genes and known annotated transposable elements were organized across samples in matrix form then imported into R (https://cran.r-project.org/). In R, counts were corrected using the counts per million procedure (cpm), pedestalled by 2, Log2 transformed, and then quantile normalized. Quality of the normalized data was challenged with no outliers detected via Tukey box plot, Pearson correlation heat map, and covariance-based Principal Component Analysis (PCA) scatterplot. Lowess modelling of the normalized data by sample type (Coefficient of Variation ~ Mean) was then performed and the fits over plotted for inspection. The lowest mean expression value across the fits at which the linear relationship with Coefficient of Variation was grossly lost was defined as the noise threshold for the data. Annotated genes and elements not having a value greater than this threshold for at least one sample were discarded as noise-biased. Surviving genes and/or elements having a value less than the threshold were floored to equal the threshold and tested for differences across sample types using the one factor Analysis of Variance (ANOVA) test under Benjamini Hochberg (BH) False Discovery Rate (FDR) Multiple Comparison Correction (MCC) condition. Annotated genes and/or elements having a corrected *p*-value < 0.05 by this test were further post hoc tested using Tukey’s Honest Significant Difference (TukeyHSD) method. Genes and/or elements having a p-value < 0.05 by this test for a specific comparison of sample types that also have a linear fold difference of means > = 1.5X were deemed to be those having differential expression between the two sample types respectively. Final summarization of sample type relationships using the union set of differentially expressed genes and/or elements was accomplished via clustered heatmap using the heatmap.2 function.

### Multiplex cytokine/chemokine immunoassay

Magnetic beads-based BioPlex Multiplex immunoassays (Bio-Rad) for human chemokine and cytokine detection were performed with THP1 and MDMs filtered culture supernatants according to manufacturer instructions. Protein concentrations were determined using serial dilutions of protein standards. Data were acquired on Bio-Plex 200 (Bio-Rad) instrument and analyzed by Bio-Plex Manager v.6 Software.

### Detection of NF-κB-activation and IFN-I induction using THP1-dual cells

Reporter THP1-Dual and THP1-Dual KO-IFNAR2 cells (InvivoGen) were cultured in 96-well tissue culture plates (0.1x10^6^ cells per 100 μl per well) in Test-RPMI-1640 medium (without Normocin) for 24 h. Then the equal volumes of culture supernatants from irradiated or poly(I:C)transfected THP1 cells (48 h post-exposure/transfection) were added into each well. The cells were incubated for 18 h, then the culture supernatants were harvested and subjected to analysis of SEAP (NF-κB-activated expression) and luciferase activity (IFNAR signaling) according to the manufacturer protocol.

### Pull-down assay

Cell pellets were washed with DPBS and then divided to two equal parts, one for IP and post-IP Western Blots and another for IP and following RNA analysis (RIP). In the samples for RIP the protein-RNA complexes were double crosslinked: with 2mM disuccinimidyl glutarate (Thermo Fisher Scientific) in DPBS for 30 min at RT with rotation, and following a wash with DPBS, crosslinked with 4% buffered paraformaldehyde (FD NeuroTechnologies) for 20 min at room temperature. After addition of Tris-HCl buffer pH 8.0 to 20mM final concentration and washing with DPBS, the cell pellets were lysed in cold IP Lysis/Wash buffer from Pierce Classic magnetic IP/co-IP kit supplemented with 1x Halt Protease Inhibitors and 0.15U/μl RNase inhibitor (all from Thermo Fisher Scientific). Magnetic beads were pre-blocked with 1mg/mL salmon testes DNA (Millipore Sigma) and 10mg/mL of BSA in Pierce IP lysis/ wash buffer for 2h at RT. A 500-μg aliquot of total protein was taken for each IP reaction, whereas 1/10 of total normalized protein extracts was taken for Western Blot control with β-actin antibody and for input control. Preparation of immune complexes, immunoprecipitation and low-pH elution was performed according to manufacturer‘s protocol.

For dsRNA IP, total RNA from 5 Gy-irradiated (48h) and control THP1 cells was isolated using Trizol, treated with DNAse I and re-purified with RNeasy columns (Qiagen). A 100-ng aliquot (0.1% of total RNA) was used for RT-qPCR reaction as input control. DsRNA was immunoprecipitated from 100 μg of purified RNA samples by overnight incubation with anti-dsRNA antibodies rJ2 (1:20) and 9D5 (1:10) (both from Millipore Sigma) at 4°C in 50 mM Tris-HCl buffer, pH 7.5 containing 40U/reaction of RNAse-OUT (Thermo Scientific). Protein A/G magnetic beads from Pierce Classic Magnetic IP/Co-IP kit (Thermo Scientific) were pre-blocked with 10 mg/ml of BSA in IP wash/lysis buffer for 2h at room temperature with rotation, washed, and then incubated with 500 μl of RNA/anti-dsRNA antibody mixtures in IP wash/lysis buffer for 2 h at 4°C with rotating. Then the beads were magnetically separated, washed 3 times with cold IP lysis/wash buffer and finally with 500 μl of cold ddH_2_O and transferred into Trizol reagent containing 20 μg/ml glycogen. Purified RNA from RIP complexes was reverse transcribed and analyzed by qPCR. Bound/input RNA ratio for each sample was calculated.

### SDS-PAGE and immunoblotting

Whole-cell extracts in RIPA buffer or Pierce IP Lysis/Wash buffer with 1x Halt protease inhibitor cocktail (Thermo Fisher Scientific) and phosphatase inhibitors (for phospho-proteins) were normalized by the total protein count using the Pierce BCA Protein Assay (Thermo Fisher Scientific). Normalized cell extracts were resuspended in Laemmli buffer (Bio-Rad) heated at 70°C for 10 min (for TLRs) or at 95°C for 5 min (for all other proteins) and spun down at 15,000 x g for 1 min. Equal amounts of protein (20–40 μg) were loaded onto 4–20% Tris-Glycine-SDS gels. Immunocomplexes eluted from magnetic beads in the pull-down assay were resuspended 4:1 in Lane Marker sample buffer (Thermo Fisher Scientific) containing 50 nM DTT and incubated for 10 min at room temperature before loading onto the gel. Proteins were transferred onto a PVDF membrane or 0.45-nm nitrocellulose membrane (for IP complexes) at 15 mA and 4°C overnight. Gels were stained with Coomassie Brilliant Blue (Bio-Rad). Membranes were blocked with TBS containing 0.1% Tween-20 and 5% dry milk or 5% BSA and incubated overnight at 4°C with the appropriate primary antibody. Membranes were then washed 5 times, incubated with the respective HRP-conjugated secondary antibody (Cell Signaling), and after washes, with Clarity chemiluminescence (ECL) reagent (Bio-Rad). Images were obtained in ChemiDoc MP Imaging System and analyzed by ImageLab software (Bio-Rad).

### Proximity ligation assay (PLA)

The proximity-dependent assay was performed according to the Duolink PLA protocol modified by Zhang and colleagues [102] for RNA-protein binding detection. Oligonucleotide probes for sense RNA binding were designed as described in [102]. The 5’ terminal part of the probe complementary to RNA of interest was designed using online FISH probe design tool (http://prober.cshl.edu/). In the negative control probe, this region comprised M13 DNA sequence. The RNA–PLA Plus-oligonucleotide probes targeting HML-2 *env* RNA were purchased from IDT DNA Technologies. The cells for PLA were prepared according to described method [102] with minor modifications. Briefly, the cells on Poly-L-lysine covered glass coverslips were washed three times with DPBS, fixed for 20 min at room temperature with Fixation buffer (BioLegend), treated with Permeabilization buffer (eBioscience) for 1 h and washed three times with DPBS. The cells were blocked with blocking buffer (10 mM Tris-acetate, pH 7.5, 10 mM magnesium acetate, 50 mM potassium acetate, 250 mM sodium chloride, 0.25 μg/μL BSA, and 0.05% Tween 20) in the presence of 20 μg/mL salmon testes DNA (Millipore Sigma) at 4°C for 1 h. A 100-nM oligonucleotide probe specific for sense HML-2 *env* RNA was added to fresh blocking buffer, heated at 70°C for 3 min, and incubated with cell samples at 4°C overnight. After three washes with DPBS, the cells were blocked in DPBS buffer containing 0.1% Tween 20, 1% BSA and 20 μg/ml salmon sperm DNA for 1 h at room temperature, followed by washing and 1-h incubation at ~20°C in Fc receptors blocking buffer (Miltenyi Biotec). After placing into 2X SSC buffer supplemented with 0.1% Tween 20 and wash with DPBS, the cells were incubated with the primary anti-MDA-5 mouse monoclonal antibody (Cell Signaling) and incubated in the Duolink Antibody Diluent (1:5,000) overnight at 4_°_C. The Duolink *in situ* PLA anti-rabbit minus probe (Millipore Sigma) was diluted according to manufacturer’s instructions in the same diluent and applied to the samples after three 5-min washes in Wash Buffer A from Duolink *in situ* Red Mouse/Rabbit kit (Millipore Sigma). Samples were then incubated for 1 h at 37°C. The following ligation, amplification and wash steps were performed according to the manufacturer’s protocol (Millipore Sigma). After a final wash, the samples were mounted onto glass slides with Duolink *In Situ* Mounting Medium containing DAPI (Millipore Sigma) and visualized on Axio Imager M2 fluorescent microscope (Zeiss).

### Immunocytochemistry and imaging

For vital plasma membrane staining, the irradiated and control THP1 cells cultured in 24-well plates were incubated in 1 ml of RPMI-1640 complete culture medium containing 5 μl of CellBrite Green reagent (Biotium) for 30 min at 37°C and 5% CO_2_. After triplicate wash with DPBS, the cells were fixed with Fixation buffer (BioLegend) for 10 min at room temperature, washed twice with DPBS, placed in DRAQ5 (30 μM)-containing DPBS solution for nuclei staining for 20 min and then imaged. F-Actin staining with Rhodamine Phalloidin reagent (Thermo Fisher Scientific) was performed by incubation of the cells, fixed for 10 min at room temperature in Fixation Buffer (BioLegend) and permeabilized in 0.1% Triton X-100 in DPBS for 3 min, with 6.6 μM rhodamine phalloidin solution in DPBS for 20 min at room temperature. After washing, the cells were placed in DAPI containing DPBS and imaged. The images were captured by Leica AF 6000 fluorescent microscope equipped with Dfc 365 Fx camera.

### Quantification and statistical analysis

The details of the statistical analysis of experiments, including used statistical tests and number of replicates are provided in the figure legends. Statistical measurements and plotting were performed using OriginPro 2019 (OriginLab). All values in this study represent means of at least three biological replicates ±SD. Two-tailed paired *t* tests and two-way ANOVA were used to compare differences between two groups and multiple groups, respectively. For the datasets with a minimum of five biological replicates and heterogeneity of variance, a Wilcoxon signed rank test was performed for paired observations and a Mann-Whitney test for unpaired observations. Real-time PCR data were quantified and analyzed using Bio-Rad CFX Manager 3.1.

## Supporting information

S1 Fig **Evaluation of reference housekeeping genes for analysis of expression of macrophage activation markers (A and B); Increased expression of macrophage activation markers in the radiation-exposed THP1 cells (C-F).** (**A** and **B**) RNA expression of three housekeeping genes in primary MDMs (**A**) and four genes in THP1 “monocytes” (**B**) at 48 h after exposure to 5 Gy _γ_IR dose was assessed by RT-qPCR. Values are given as cycle threshold numbers (C_q_). The boxes represent the lower and upper quartiles with lines in between representing medians; whiskers represent the range of data from three technical triplicates from minimum six biological replicates. The mean values are shown in panels; NS—p>0.05, paired Wilcoxon test. (**C**-**E**) Proportion of THP1 cells presenting inflammation surface markers CD80^+^ (**C**), CD86^+^ (**D**), HLA-DR^+^ (**E**) and anti-inflammatory marker CD206 (**F**) in populations of non-irradiated (0 Gy) and irradiated (5 Gy) cells detected by flow cytometry measurement of median fluorescence intensity (MFI) of viable (DAPI^-^) cells, 72h after _γ_IR (graphs show representatives of 3 independent experiments).
(TIF)

S2 Fig Radiation-exposed monocytes THP1 obtain macrophage-like morphology. (**A**) Vital staining of THP1 cells with CellBrite cytoplasmic membrane dye (green) and nuclei-specific dye Draq5 (orange) to visualize size of the cytoplasm and nuclei in the control non-irradiated and 5Gy _γ_IR exposed cells. Scale bars: 75 μm. (**B**) Images of irradiated or control human monocytes THP1 stained with Draq5 (nuclei), captured by imaging flow cytometry. (**C**) Quantitation of the effect of 5Gy _γ_IR dose on the abundance of cytoplasmic granules in viable THP1 cells by imaging flow cytometry. Representative histograms from three biological samples are shown. (**D**) Quantitation of cytoplasmic granules in viable THP1 cells by imaging flow cytometry (Draq5 staining of nuclei). Error bars indicate ±SD of three independent biological replicates; * *p*<0.05, ** *p*<0.01, ^NS^ non-significant.
(TIF)

S3 Fig Radiation-exposed THP1 cells display increased expression of macrophage adhesion marker Integrin β1. Integrin β1^+^ cells in populations of non-irradiated, irradiated, PMA and poly(I:C)-treated THP1 detected by flow cytometry of viable (DAPI^-^) cells, 72h after _γ_IR: left panel shows ratio of integrin β1^+^ cells in the samples of unexposed and 5Gy dose exposed cells; the right panel shows quantitation data of integrin β1^+^ cells in indicated THP1 samples (flow cytometry data of viable, DAPI-negative cells were analyzed, a representative graph of three independent measurements).
(TIF)

S4 Fig Exposure to gamma radiation activates expression of interferon-stimulated genes and partially affects ssRNA cytoplasmic sensors. (**A**) Effect of dsRNA on IFNβ expression in THP1 cells, measured by RT-qPCR at 24 h after treatment. Cells were treated with poly(I:C) with or without indicated transfection reagents. Analysis of RNA abundance by RT-qPCR without reverse transcriptase reaction is shown (light-blue columns). Error bars: ±SD of 5 independent biological replicates; * *p*<0.05, paired Wilcoxon test. (**B**) Heatmap depicting expression of interferon-stimulated genes (ISG) shown in Fig 3E in THP1 cells exposed to a 5 Gy _γ_IR dose vs. non-irradiated cells, measured by PCR array of total cellular RNA samples, 48h after γIR exposure: rows: ISG codes; columns: samples. Color codes are shown on the left panel. (**C**) Scatter plot showing ISG expression in irradiated vs. control MDMs, measured by PCR array of total cellular RNA samples, 48h post-irradiation. Red dots indicate twofold increase in gene expression in irradiated cells vs non-irradiated cells. (**D**) Western blot of cytoplasmic ssRNA sensors TLR7 and TLR8 (40 μg of total protein) in THP1 cells, 48h after exposure to indicated doses of _γ_IR; β actin antibody served as a reference standard.
(TIF)

S5 Fig Gamma radiation activates HERV transcription in a dose-dependent manner. (**A**) Covariance-based clustered heatmap using the unique union set of 955 differentially expressed retroelements and ERVs identified by analysis of RNA-seq data across non-irradiated and irradiated THP1 (s1-s3) and MDMs (d1-d4), 48h after exposure. (**B**) Expression of different HERVs, identified activated in association with cancer, neurodegenerative diseases or in human embryonic cells upon differentiation [40, 42, 86–88], in THP1 cells, 48h post-irradiation. Total RNA was quantified by RT-qPCR with primers specific for 43 HERVs. Error bars: ±SD of three independent biological replicates; elements, significantly responding to _γ_IR are indicated by red symbols. (**C** and **D**) Effect of gamma radiation doses on the transcription of HERVK HML-2 env (**C**) and 5’LTR (**D**) in THP1 cells, 48h after exposure to indicated doses of _γ_IR, measured by RT-qPCR. The fold gene expression (_ΔΔ_Ct) was calculated in relation to β actin as a reference gene. Error bars indicate ±SD of three independent biological replicates. (**E**) Time course of HML-2 *env* expression in THP1 cells exposed to indicated doses of _γ_IR and measured by RT-qPCR at shown time-points. B-spline curves were plotted based on the values of fold gene expression for each time point calculated as _ΔΔ_Ct relative to β-actin. Error bars: ±SD of four independent biological replicates. (**F**) Relative count of antisense RNA, measured by RT-qPCR, of differentially expressed HERVK (sense RNA count shown in Fig 4C) in THP1 cells, 48h post-irradiation. Error bars: ±SD of three independent biological replicates. (**G**) Gamma radiation activates antisense transcription of HERVK HML-2 earlier than the sense transcription: relative RNA count, measured by RT-qPCR, of HML-2 sense and antisense *env* transcripts, 24 and 48h after irradiation of THP1 cells. In all panels, * p<0.05, ** p<0.01, ^NS^ non significant.
(TIF)

S6 Fig shRNA-induced HERVK HML-2 *env* knockdown changes expression of multiple HERV subgroups and results in reduced expression of interferon-stimulated genes. (**A**) Relative count of HERVK HML-2 *env* RNA in THP1 cells infected with lentivial vector pLKO.1 puro expressing indicated shRNA, selected with 0.5 μg/ml puromycin. RNA was isolated and relative *env* RNA abundance was measured by RT-qPCR. The fold change RNA count (_ΔΔ_Ct) was calculated in relation to β actin reference gene. Error bars indicate ±SD of three independent biological replicates. (**B**) Transcription, measured by RT-qPCR, of randomly selected differentially expressed HERVK proviruses, identified by transcriptomic analysis, in THP1 cells expressing control (grey) or shRNA-Env (red), 48h post-irradiation. In panels A and B, error bars: ±SD of three independent biological replicates; * *p*<0.05, ** *p*<0.01, two-tailed paired *t* test. (**C**) Ratio of HML-2 RNA bound to anti-dsRNA antibodies to RNA input. RT-qPCR of RNA IP complexes with rJ2 and 9D5 antibodies, 48h post-irradiation. Error bars: ±SD of five independent biological replicates; * p<0.05, paired Wilcoxon test. (**D**) Heatmap depicting expression of interferon-stimulated genes (ISG) shown on Fig 5E in THP1 cells exposed to 5Gy _γ_IR, 48h post-exposure: cells expressing shRNA-Env vs control shRNA, measured by PCR array of total cellular RNA samples. Rows: ISG codes; columns: samples. Color codes are shown on the left panel.
(TIF)

S1 Table Differentially transcribed retroelements. **(A)** Differentially transcribed retroelements in THP1 monocytes: 5Gy vs 0Gy; **(B)** Differentially transcribed retroelements in primary human MDM: 5Gy vs 0Gy; **(C)** Differentially transcribed retroelements: Common in THP1 & MDM: 5Gy vs 0Gy; **(D)** Differentially transcribed HERVK in THP1 monocytes: 5Gy vs 0Gy; **(E)** Differentially transcribed HERVK in primary human MDM: 5Gy vs 0Gy.
(XLSX)

S2 Table Regions in human genome complementary to HERVK HML-2 env shRNAs with or without one mismatch. **(A)** Complementary regions to shRNA-Env1; **(B)** Complementary regions to shRNA-Env1070.
(XLSX)

## References

[1] Bentzen SM. Preventing or reducing late side effects of radiation therapy: radiobiology meets molecular pathology. Nat Rev Cancer 2006;6(9):702–13. 10.1038/nrc1950 . 16929324

[2] Lauber K, Ernst A, Orth M, Herrmann M, Belka C. Dying cell clearance and its impact on the outcome of tumor radiotherapy. Front Oncol 2012;2:116 10.3389/fonc.2012.00116 22973558 PMC3438527

[3] Vacchelli E, Vitale I, Tartour E, Eggermont A, Sautes-Fridman C, Galon J, et al Trial Watch: Anticancer radioimmunotherapy. Oncoimmunology. 2013;2(9):e25595 10.4161/onci.25595 24319634 PMC3850274

[4] Teresa Pinto A, Laranjeiro Pinto M, Patricia Cardoso A, Monteiro C, Teixeira Pinto M, Filipe Maia A, et al Ionizing radiation modulates human macrophages towards a pro-inflammatory phenotype preserving their pro-invasive and pro-angiogenic capacities. Sci Rep. 2016;6:18765 10.1038/srep18765 26735768 PMC4702523

[5] Mantovani A, Sica A. Macrophages, innate immunity and cancer: balance, tolerance, and diversity. Curr Opin Immunol 2010;22(2):231–7. Epub 2010/02/11. 10.1016/j.coi.2010.01.009 . 20144856

[6] Martinez FO, Gordon S. The M1 and M2 paradigm of macrophage activation: time for reassessment. F1000Prime Rep. 2014;6:13 Epub 2014/03/29. 10.12703/P6-13 24669294 PMC3944738

[7] Gandhi S, Chandna S. Radiation-induced inflammatory cascade and its reverberating crosstalks as potential cause of post-radiotherapy second malignancies. Cancer Metastasis Rev 2017;36(2):375–93. 10.1007/s10555-017-9669-x . 28707199

[8] Leblond MM, Peres EA, Helaine C, Gerault AN, Moulin D, Anfray C, et al M2 macrophages are more resistant than M1 macrophages following radiation therapy in the context of glioblastoma. Oncotarget. 2017;8(42):72597–612. Epub 2017/10/27. 10.18632/oncotarget.19994 29069812 PMC5641155

[9] Kozin SV, Kamoun WS, Huang Y, Dawson MR, Jain RK, Duda DG. Recruitment of myeloid but not endothelial precursor cells facilitates tumor regrowth after local irradiation. Cancer Res 2010;70(14):5679–85. 10.1158/0008-5472.CAN-09-4446 20631066 PMC2918387

[10] Barker HE, Paget JT, Khan AA, Harrington KJ. The tumour microenvironment after radiotherapy: mechanisms of resistance and recurrence. Nat Rev Cancer 2015;15(7):409–25. 10.1038/nrc3958 26105538 PMC4896389

[11] Prakash H, Klug F, Nadella V, Mazumdar V, Schmitz-Winnenthal H, Umansky L. Low doses of gamma irradiation potentially modifies immunosuppressive tumor microenvironment by retuning tumor-associated macrophages: lesson from insulinoma. Carcinogenesis 2016;37(3):301–13. 10.1093/carcin/bgw007 . 26785731

[12] Shi X, Shiao SL. The role of macrophage phenotype in regulating the response to radiation therapy. Transl Res. 2018;191:64–80. 10.1016/j.trsl.2017.11.002 29175267 PMC6018060

[13] Loinard C, Vilar J, Milliat F, Levy B, Benderitter M. Monocytes/Macrophages Mobilization Orchestrate Neovascularization after Localized Colorectal Irradiation. Radiat Res 2017;187(5):549–61. Epub 2017/03/21. 10.1667/RR14398.1 . 28319461

[14] Mathy NW, Chen XM. Long non-coding RNAs (lncRNAs) and their transcriptional control of inflammatory responses. J Biol Chem 2017;292(30):12375–82. 10.1074/jbc.R116.760884 28615453 PMC5535013

[15] Szabo A, Rajnavolgyi E. Collaboration of Toll-like and RIG-I-like receptors in human dendritic cells: tRIGgering antiviral innate immune responses. American journal of clinical and experimental immunology 2013;2(3):195–207. 24179728 PMC3808934

[16] Hurst TP, Magiorkinis G. Activation of the innate immune response by endogenous retroviruses. J Gen Virol 2015;96(Pt 6):1207–18. 10.1099/jgv.0.000017 . 26068187

[17] Huang Z, Luo Q, Yao F, Qing C, Ye J, Deng Y, et al Identification of Differentially Expressed Long Non-coding RNAs in Polarized Macrophages. Sci Rep. 2016;6:19705 10.1038/srep19705 26796525 PMC4726337

[18] Mu X, Ahmad S, Hur S. Endogenous Retroelements and the Host Innate Immune Sensors. Adv Immunol 2016;132:47–69. 10.1016/bs.ai.2016.07.001 27769507 PMC5135014

[19] Jacobs BL, Langland JO. When two strands are better than one: the mediators and modulators of the cellular responses to double-stranded RNA. Virology 1996;219(2):339–49. Epub 1996/05/15. 10.1006/viro.1996.0259 . 8638399

[20] Dempoya J, Matsumiya T, Imaizumi T, Hayakari R, Xing F, Yoshida H, et al Double-stranded RNA induces biphasic STAT1 phosphorylation by both type I interferon (IFN)-dependent and type I IFN-independent pathways. J Virol. 2012;86(23):12760–9. Epub 2012/09/14. 10.1128/JVI.01881-12 22973045 PMC3497619

[21] Sampey GC, Saifuddin M, Schwab A, Barclay R, Punya S, Chung MC, et al Exosomes from HIV-1-infected Cells Stimulate Production of Pro-inflammatory Cytokines through Trans-activating Response (TAR) RNA. J Biol Chem. 2016;291(3):1251–66. 10.1074/jbc.M115.662171 26553869 PMC4714213

[22] Sohn J, Hur S. Filament assemblies in foreign nucleic acid sensors. Curr Opin Struct Biol 2016;37:134–44. 10.1016/j.sbi.2016.01.011 26859869 PMC5070476

[23] Saha SK, Pietras EM, He JQ, Kang JR, Liu SY, Oganesyan G, et al Regulation of antiviral responses by a direct and specific interaction between TRAF3 and Cardif. EMBO J. 2006;25(14):3257–63. 10.1038/sj.emboj.7601220 16858409 PMC1523175

[24] Pichlmair A, Schulz O, Tan CP, Naslund TI, Liljestrom P, Weber F, et al RIG-I-mediated antiviral responses to single-stranded RNA bearing 5’-phosphates. Science. 2006;314(5801):997–1001. 10.1126/science.1132998 . 17038589

[25] Kato H, Takeuchi O, Mikamo-Satoh E, Hirai R, Kawai T, Matsushita K, et al Length-dependent recognition of double-stranded ribonucleic acids by retinoic acid-inducible gene-I and melanoma differentiation-associated gene 5. J Exp Med. 2008;205(7):1601–10. 10.1084/jem.20080091 18591409 PMC2442638

[26] Strick R, Strissel PL, Baylin SB, Chiappinelli KB. Unraveling the molecular pathways of DNA-methylation inhibitors: human endogenous retroviruses induce the innate immune response in tumors. Oncoimmunology. 2016;5(5):e1122160 10.1080/2162402X.2015.1122160 27467919 PMC4910697

[27] Yu Q, Katlinskaya YV, Carbone CJ, Zhao B, Katlinski KV, Zheng H, et al DNA-damage-induced type I interferon promotes senescence and inhibits stem cell function. Cell Rep. 2015;11(5):785–97. Epub 2015/04/30. 10.1016/j.celrep.2015.03.069 25921537 PMC4426031

[28] Katlinskaya YV, Katlinski KV, Yu Q, Ortiz A, Beiting DP, Brice A, et al Suppression of Type I Interferon Signaling Overcomes Oncogene-Induced Senescence and Mediates Melanoma Development and Progression. Cell Rep. 2016;15(1):171–80. Epub 2016/04/08. 10.1016/j.celrep.2016.03.006 27052162 PMC4826807

[29] Billiau A. Anti-inflammatory properties of Type I interferons. Antivir Res 2006;71(2–3):108–16. 10.1016/j.antiviral.2006.03.006 . 16626815 PMC7114336

[30] Chang EY, Guo B, Doyle SE, Cheng G. Cutting edge: involvement of the type I IFN production and signaling pathway in lipopolysaccharide-induced IL-10 production. J Immunol 2007;178(11):6705–9. 10.4049/jimmunol.178.11.6705 . 17513714

[31] Benveniste EN, Qin H. Type I interferons as anti-inflammatory mediators. Sci STKE. 2007;2007(416):pe70 10.1126/stke.4162007pe70 . 18073382

[32] Wilson EB, Yamada DH, Elsaesser H, Herskovitz J, Deng J, Cheng G, et al Blockade of chronic type I interferon signaling to control persistent LCMV infection. Science. 2013;340(6129):202–7. 10.1126/science.1235208 23580528 PMC3704950

[33] Honke N, Shaabani N, Merches K, Gassa A, Kraft A, Ehrhardt K, et al Immunoactivation induced by chronic viral infection inhibits viral replication and drives immunosuppression through sustained IFN-I responses. Eur J Immunol. 2016;46(2):372–80. 10.1002/eji.201545765 26507703 PMC5063111

[34] Ivashkiv LB, Donlin LT. Regulation of type I interferon responses. Nat Rev Immunol 2014;14(1):36–49. 10.1038/nri3581 24362405 PMC4084561

[35] Bannert N, Kurth R. Retroelements and the human genome: new perspectives on an old relation. Proc Natl Acad Sci U S A 2004;101 Suppl 2:14572–9 10.1073/pnas.0404838101 15310846 PMC521986

[36] Buzdin AA, Prassolov V, Garazha AV. Friends-Enemies: Endogenous Retroviruses Are Major Transcriptional Regulators of Human DNA. Front Chem 2017;5:35 10.3389/fchem.2017.00035 28642863 PMC5462908

[37] Yu P, Lubben W, Slomka H, Gebler J, Konert M, Cai C, et al Nucleic acid-sensing Toll-like receptors are essential for the control of endogenous retrovirus viremia and ERV-induced tumors. Immunity. 2012;37(5):867–79. 10.1016/j.immuni.2012.07.018 . 23142781

[38] Volkman HE, Stetson DB. The enemy within: endogenous retroelements and autoimmune disease. Nat Immunol 2014;15(5):415–22. 10.1038/ni.2872 24747712 PMC4131434

[39] Hung T, Pratt GA, Sundararaman B, Townsend MJ, Chaivorapol C, Bhangale T, et al The Ro60 autoantigen binds endogenous retroelements and regulates inflammatory gene expression. Science. 2015;350(6259):455–9. 10.1126/science.aac7442 26382853 PMC4691329

[40] Chiappinelli KB, Strissel PL, Desrichard A, Li H, Henke C, Akman B, et al Inhibiting DNA Methylation Causes an Interferon Response in Cancer via dsRNA Including Endogenous Retroviruses. Cell. 2015;162(5):974–86. 10.1016/j.cell.2015.07.011 26317466 PMC4556003

[41] Chuong EB, Elde NC, Feschotte C. Regulatory evolution of innate immunity through co-option of endogenous retroviruses. Science 2016;351(6277):1083–7. 10.1126/science.aad5497 26941318 PMC4887275

[42] Canadas I, Thummalapalli R, Kim JW, Kitajima S, Jenkins RW, Christensen CL, et al Tumor innate immunity primed by specific interferon-stimulated endogenous retroviruses. Nat Med. 2018;24(8):1143–50. 10.1038/s41591-018-0116-5 30038220 PMC6082722

[43] Iyer G, Wang AR, Brennan SR, Bourgeois S, Armstrong E, Shah P, et al Identification of stable housekeeping genes in response to ionizing radiation in cancer research. Sci Rep. 2017;7:43763 Epub 2017/03/07. 10.1038/srep43763 28262749 PMC5338320

[44] Verreck FA, de Boer T, Langenberg DM, Hoeve MA, Kramer M, Vaisberg E, et al Human IL-23-producing type 1 macrophages promote but IL-10-producing type 2 macrophages subvert immunity to (myco)bacteria. Proc Natl Acad Sci U S A. 2004;101(13):4560–5. 10.1073/pnas.0400983101 15070757 PMC384786

[45] Jaguin M, Houlbert N, Fardel O, Lecureur V. Polarization profiles of human M-CSF-generated macrophages and comparison of M1-markers in classically activated macrophages from GM-CSF and M-CSF origin. Cell Immunol 2013;281(1):51–61. 10.1016/j.cellimm.2013.01.010 . 23454681

[46] Tsang JS, Schwartzberg PL, Kotliarov Y, Biancotto A, Xie Z, Germain RN, et al Global analyses of human immune variation reveal baseline predictors of postvaccination responses. Cell. 2014;157(2):499–513. Epub 2014/04/15. 10.1016/j.cell.2014.03.031 24725414 PMC4139290

[47] Buscher K, Ehinger E, Gupta P, Pramod AB, Wolf D, Tweet G, et al Natural variation of macrophage activation as disease-relevant phenotype predictive of inflammation and cancer survival. Nat Commun. 2017;8:16041 Epub 2017/07/25. 10.1038/ncomms16041 28737175 PMC5527282

[48] Wynn TA, Chawla A, Pollard JW. Macrophage biology in development, homeostasis and disease. Nature. 2013;496(7446):445–55. Epub 2013/04/27. 10.1038/nature12034 23619691 PMC3725458

[49] Tomita K, Freeman BL, Bronk SF, LeBrasseur NK, White TA, Hirsova P, et al CXCL10-Mediates Macrophage, but not Other Innate Immune Cells-Associated Inflammation in Murine Nonalcoholic Steatohepatitis. Sci Rep. 2016;6:28786 Epub 2016/06/29. 10.1038/srep28786 27349927 PMC4923862

[50] Liu P, Jia S, Lou Y, He K, Xu LX. Cryo-thermal therapy inducing MI macrophage polarization created CXCL10 and IL-6-rich pro-inflammatory environment for CD4(+) T cell-mediated anti-tumor immunity. Int J Hyperth 2019;36(1):408–20. Epub 2019/03/21. 10.1080/02656736.2019.1579373 . 30892102

[51] Basheeruddin K, Rechtoris C, Mazzone T. Evaluation of the role of Ap1-like proteins in the enhanced apolipoprotein E gene transcription accompanying phorbol ester induced macrophage differentiation. Biochim Biophys Acta 1994;1218(2):235–41. Epub 1994/06/21. 10.1016/0167-4781(94)90021-3 . 8018731

[52] Schwende H, Fitzke E, Ambs P, Dieter P. Differences in the state of differentiation of THP-1 cells induced by phorbol ester and 1,25-dihydroxyvitamin D3. J Leukoc Biol. 1996;59(4):555–61. Epub 1996/04/01. . 8613704

[53] Barilli A, Rotoli BM, Visigalli R, Bussolati O, Gazzola GC, Dall’Asta V. Arginine transport in human monocytic leukemia THP-1 cells during macrophage differentiation. J Leukoc Biol 2011;90(2):293–303. Epub 2011/05/19. 10.1189/jlb.0910510 . 21586674

[54] Helm O, Held-Feindt J, Schafer H, Sebens S. M1 and M2: there is no "good" and "bad"-How macrophages promote malignancy-associated features in tumorigenesis. Oncoimmunology. 2014;3(7):e946818 10.4161/21624011.2014.946818 25610733 PMC4292215

[55] Nawaz A, Aminuddin A, Kado T, Takikawa A, Yamamoto S, Tsuneyama K, et al CD206(+) M2-like macrophages regulate systemic glucose metabolism by inhibiting proliferation of adipocyte progenitors. Nat Commun. 2017;8(1):286 10.1038/s41467-017-00231-1 28819169 PMC5561263

[56] de Bruin RG, Shiue L, Prins J, de Boer HC, Singh A, Fagg WS, et al Quaking promotes monocyte differentiation into pro-atherogenic macrophages by controlling pre-mRNA splicing and gene expression. Nat Commun. 2016;7:10846 Epub 2016/04/01. 10.1038/ncomms10846 27029405 PMC4821877

[57] Lee JJ, Kim BC, Park MJ, Lee YS, Kim YN, Lee BL, et al PTEN status switches cell fate between premature senescence and apoptosis in glioma exposed to ionizing radiation. Cell Death Differ. 2011;18(4):666–77. Epub 2010/11/13. 10.1038/cdd.2010.139 21072054 PMC3131905

[58] Li M, You L, Xue J, Lu Y. Ionizing Radiation-Induced Cellular Senescence in Normal, Non-transformed Cells and the Involved DNA Damage Response: A Mini Review. Front Pharmacol. 2018;9:522 Epub 2018/06/07. 10.3389/fphar.2018.00522 29872395 PMC5972185

[59] Kuilman T, Michaloglou C, Vredeveld LC, Douma S, van Doorn R, Desmet CJ, et al Oncogene-induced senescence relayed by an interleukin-dependent inflammatory network. Cell. 2008;133(6):1019–31. Epub 2008/06/17. 10.1016/j.cell.2008.03.039 . 18555778

[60] Freund A, Orjalo AV, Desprez PY, Campisi J. Inflammatory networks during cellular senescence: causes and consequences. Trends Mol Med. 2010;16(5):238–46. Epub 2010/05/07. 10.1016/j.molmed.2010.03.003 20444648 PMC2879478

[61] Kojima H, Inoue T, Kunimoto H, Nakajima K. IL-6-STAT3 signaling and premature senescence. JAKSTAT. 2013;2(4):e25763 Epub 2014/01/15. 10.4161/jkst.25763 24416650 PMC3876432

[62] Maciel-Baron LA, Moreno-Blas D, Morales-Rosales SL, Gonzalez-Puertos VY, Lopez-Diazguerrero NE, Torres C, et al Cellular Senescence, Neurological Function, and Redox State. Antioxid Redox Signal. 2018;28(18):1704–23. Epub 2017/05/04. 10.1089/ars.2017.7112 . 28467755

[63] Fang L, Igarashi M, Leung J, Sugrue MM, Lee SW, Aaronson SA. p21Waf1/Cip1/Sdi1 induces permanent growth arrest with markers of replicative senescence in human tumor cells lacking functional p53. Oncogene. 1999;18(18):2789–97. Epub 1999/06/11. 10.1038/sj.onc.1202615 . 10362249

[64] Passos JF, Nelson G, Wang C, Richter T, Simillion C, Proctor CJ, et al Feedback between p21 and reactive oxygen production is necessary for cell senescence. Mol Syst Biol. 2010;6:347 Epub 2010/02/18. 10.1038/msb.2010.5 20160708 PMC2835567

[65] Dimri GP, Lee X, Basile G, Acosta M, Scott G, Roskelley C, et al A biomarker that identifies senescent human cells in culture and in aging skin in vivo. Proc Natl Acad Sci U S A. 1995;92(20):9363–7. Epub 1995/09/26. 10.1073/pnas.92.20.9363 7568133 PMC40985

[66] Hall BM, Balan V, Gleiberman AS, Strom E, Krasnov P, Virtuoso LP, et al p16(Ink4a) and senescence-associated beta-galactosidase can be induced in macrophages as part of a reversible response to physiological stimuli. Aging (Albany NY). 2017;9(8):1867–84. Epub 2017/08/05. 10.18632/aging.101268 28768895 PMC5611982

[67] Buchan JR, Parker R. Eukaryotic stress granules: the ins and outs of translation. Mol Cell. 2009;36(6):932–41. Epub 2010/01/13. 10.1016/j.molcel.2009.11.020 20064460 PMC2813218

[68] Castano D, Barrera LF, Rojas M. Mycobacterium tuberculosis alters the differentiation of monocytes into macrophages in vitro. Cell Immunol 2011;268(2):60–7. 10.1016/j.cellimm.2011.02.006 . 21420074

[69] Ammon C, Meyer SP, Schwarzfischer L, Krause SW, Andreesen R, Kreutz M. Comparative analysis of integrin expression on monocyte-derived macrophages and monocyte-derived dendritic cells. Immunology. 2000;100(3):364–9. 10.1046/j.1365-2567.2000.00056.x 10929059 PMC2327027

[70] Cao C, Lawrence DA, Strickland DK, Zhang L. A specific role of integrin Mac-1 in accelerated macrophage efflux to the lymphatics. Blood. 2005;106(9):3234–41. 10.1182/blood-2005-03-1288 16002427 PMC1895336

[71] Podolnikova NP, Kushchayeva YS, Wu Y, Faust J, Ugarova TP. The Role of Integrins alphaMbeta2 (Mac-1, CD11b/CD18) and alphaDbeta2 (CD11d/CD18) in Macrophage Fusion. Am J Pathol. 2016;186(8):2105–16. Epub 2016/06/19. 10.1016/j.ajpath.2016.04.001 27315778 PMC4973655

[72] Wang N, Liang H, Zen K. Molecular mechanisms that influence the macrophage m1-m2 polarization balance. Frontiers in immunology. 2014;5:614 10.3389/fimmu.2014.00614 25506346 PMC4246889

[73] Wu Q, Allouch A, Martins I, Modjtahedi N, Deutsch E, Perfettini JL. Macrophage biology plays a central role during ionizing radiation-elicited tumor response. Biomed J. 2017;40(4):200–11. 10.1016/j.bj.2017.06.003 28918908 PMC6136289

[74] Mackenzie KJ, Carroll P, Martin CA, Murina O, Fluteau A, Simpson DJ, et al cGAS surveillance of micronuclei links genome instability to innate immunity. Nature. 2017;548(7668):461–5. Epub 2017/07/25. 10.1038/nature23449 28738408 PMC5870830

[75] Harding SM, Benci JL, Irianto J, Discher DE, Minn AJ, Greenberg RA. Mitotic progression following DNA damage enables pattern recognition within micronuclei. Nature. 2017;548(7668):466–70. Epub 2017/08/02. 10.1038/nature23470 28759889 PMC5857357

[76] Erdal E, Haider S, Rehwinkel J, Harris AL, McHugh PJ. A prosurvival DNA damage-induced cytoplasmic interferon response is mediated by end resection factors and is limited by Trex1. Genes Dev. 2017;31(4):353–69. Epub 2017/03/11. 10.1101/gad.289769.116 28279982 PMC5358756

[77] Fleetwood AJ, Dinh H, Cook AD, Hertzog PJ, Hamilton JA. GM-CSF- and M-CSF-dependent macrophage phenotypes display differential dependence on type I interferon signaling. J Leukoc Biol 2009;86(2):411–21. 10.1189/jlb.1108702 . 19406830

[78] Dimberg A, Karlberg I, Nilsson K, Oberg F. Ser727/Tyr701-phosphorylated Stat1 is required for the regulation of c-Myc, cyclins, and p27Kip1 associated with ATRA-induced G0/G1 arrest of U-937 cells. Blood 2003;102(1):254–61. 10.1182/blood-2002-10-3149 . 12637327

[79] Sadzak I, Schiff M, Gattermeier I, Glinitzer R, Sauer I, Saalmuller A, et al Recruitment of Stat1 to chromatin is required for interferon-induced serine phosphorylation of Stat1 transactivation domain. Proc Natl Acad Sci U S A. 2008;105(26):8944–9. 10.1073/pnas.0801794105 18574148 PMC2435588

[80] Hipp MM, Shepherd D, Gileadi U, Aichinger MC, Kessler BM, Edelmann MJ, et al Processing of human toll-like receptor 7 by furin-like proprotein convertases is required for its accumulation and activity in endosomes. Immunity. 2013;39(4):711–21. Epub 2013/10/22. 10.1016/j.immuni.2013.09.004 24138882 PMC4839496

[81] Slater L, Bartlett NW, Haas JJ, Zhu J, Message SD, Walton RP, et al Co-ordinated role of TLR3, RIG-I and MDA5 in the innate response to rhinovirus in bronchial epithelium. PLoS Pathog. 2010;6(11):e1001178 Epub 2010/11/17. 10.1371/journal.ppat.1001178 21079690 PMC2973831

[82] Seth RB, Sun L, Ea CK, Chen ZJ. Identification and characterization of MAVS, a mitochondrial antiviral signaling protein that activates NF-kappaB and IRF 3. Cell 2005;122(5):669–82. Epub 2005/08/30. 10.1016/j.cell.2005.08.012 . 16125763

[83] Scott I, Norris KL. The mitochondrial antiviral signaling protein, MAVS, is cleaved during apoptosis. Biochem Biophys Res Commun. 2008;375(1):101–6. Epub 2008/08/12. 10.1016/j.bbrc.2008.07.147 18692023 PMC2600422

[84] Wang Z, Ji J, Peng D, Ma F, Cheng G, Qin FX. Complex Regulation Pattern of IRF3 Activation Revealed by a Novel Dimerization Reporter System. J Immunol 2016;196(10):4322–30. Epub 2016/04/06. 10.4049/jimmunol.1502458 . 27045107

[85] Montesion M, Bhardwaj N, Williams ZH, Kuperwasser C, Coffin JM. Mechanisms of HERV-K (HML-2) Transcription during Human Mammary Epithelial Cell Transformation. J Virol. 2018;92(1). Epub 2017/10/20. 10.1128/JVI.01258-17 29046454 PMC5730778

[86] Laska MJ, Brudek T, Nissen KK, Christensen T, Moller-Larsen A, Petersen T, et al Expression of HERV-Fc1, a human endogenous retrovirus, is increased in patients with active multiple sclerosis. J Virol. 2012;86(7):3713–22. Epub 2012/01/27. 10.1128/JVI.06723-11 22278236 PMC3302483

[87] Grow EJ, Flynn RA, Chavez SL, Bayless NL, Wossidlo M, Wesche DJ, et al Intrinsic retroviral reactivation in human preimplantation embryos and pluripotent cells. Nature. 2015;522(7555):221–5. Epub 2015/04/22. 10.1038/nature14308 25896322 PMC4503379

[88] Li W, Lee MH, Henderson L, Tyagi R, Bachani M, Steiner J, et al Human endogenous retrovirus-K contributes to motor neuron disease. Science translational medicine. 2015;7(307):307ra153 10.1126/scitranslmed.aac8201 . 26424568 PMC6344353

[89] Subramanian RP, Wildschutte JH, Russo C, Coffin JM. Identification, characterization, and comparative genomic distribution of the HERV-K (HML-2) group of human endogenous retroviruses. Retrovirology 2011;8:90 10.1186/1742-4690-8-90 22067224 PMC3228705

[90] Lybecker M, Zimmermann B, Bilusic I, Tukhtubaeva N, Schroeder R. The double-stranded transcriptome of Escherichia coli. Proc Natl Acad Sci U S A. 2014;111(8):3134–9. Epub 2014/01/24. 10.1073/pnas.1315974111 24453212 PMC3939876

[91] Zapata JC, Campilongo F, Barclay RA, DeMarino C, Iglesias-Ussel MD, Kashanchi F, et al The Human Immunodeficiency Virus 1 ASP RNA promotes viral latency by recruiting the Polycomb Repressor Complex 2 and promoting nucleosome assembly. Virology. 2017;506:34–44. 10.1016/j.virol.2017.03.002 28340355 PMC5505171

[92] Yoshida T, Naito Y, Sasaki K, Uchida E, Sato Y, Naito M, et al Estimated number of off-target candidate sites for antisense oligonucleotides in human mRNA sequences. Genes Cells. 2018;23(6):448–55. Epub 2018/04/19. 10.1111/gtc.12587 . 29667281

[93] De Filippo K, Dudeck A, Hasenberg M, Nye E, van Rooijen N, Hartmann K, et al Mast cell and macrophage chemokines CXCL1/CXCL2 control the early stage of neutrophil recruitment during tissue inflammation. Blood. 2013;121(24):4930–7. Epub 2013/05/07. 10.1182/blood-2013-02-486217 . 23645836

[94] Luster AD, Ravetch JV. Biochemical characterization of a gamma interferon-inducible cytokine (IP-10). J Exp Med. 1987;166(4):1084–97. Epub 1987/10/01. 10.1084/jem.166.4.1084 2443596 PMC2188708

[95] Liu M, Guo S, Hibbert JM, Jain V, Singh N, Wilson NO, et al CXCL10/IP-10 in infectious diseases pathogenesis and potential therapeutic implications. Cytokine Growth Factor Rev. 2011;22(3):121–30. Epub 2011/08/02. 10.1016/j.cytogfr.2011.06.001 21802343 PMC3203691

[96] Roulois D, Loo Yau H, Singhania R, Wang Y, Danesh A, Shen SY, et al DNA-Demethylating Agents Target Colorectal Cancer Cells by Inducing Viral Mimicry by Endogenous Transcripts. Cell. 2015;162(5):961–73. 10.1016/j.cell.2015.07.056 26317465 PMC4843502

[97] Cipriani C, Ricceri L, Matteucci C, De Felice A, Tartaglione AM, Argaw-Denboba A, et al High expression of Endogenous Retroviruses from intrauterine life to adulthood in two mouse models of Autism Spectrum Disorders. Sci Rep. 2018;8(1):629 Epub 2018/01/14. 10.1038/s41598-017-19035-w 29330412 PMC5766538

[98] Smith CC, Beckermann KE, Bortone DS, De Cubas AA, Bixby LM, Lee SJ, et al Endogenous retroviral signatures predict immunotherapy response in clear cell renal cell carcinoma. J Clin Invest. 2018;128(11):4804–20. Epub 2018/08/24. 10.1172/JCI121476 30137025 PMC6205406

[99] Schmidt N, Domingues P, Golebiowski F, Patzina C, Tatham MH, Hay RT, et al An influenza virus-triggered SUMO switch orchestrates co-opted endogenous retroviruses to stimulate host antiviral immunity. Proc Natl Acad Sci U S A. 2019;116(35):17399–408. Epub 2019/08/09. 10.1073/pnas.1907031116 . 31391303 PMC6717285

[100] Iordanskiy S, Berro R, Altieri M, Kashanchi F, Bukrinsky M. Intracytoplasmic maturation of the human immunodeficiency virus type 1 reverse transcription complexes determines their capacity to integrate into chromatin. Retrovirology. 2006;3:4 Epub 2006/01/18. 10.1186/1742-4690-3-4 . 16409631 PMC1360674

[101] Iordanskiy SN, Bukrinsky MI. Analysis of Viral and Cellular Proteins in HIV-1 Reverse Transcription Complexes by Co-immunoprecipitation. Methods Mol Biol 2009;485:121–34. Epub 2008/11/21. 10.1007/978-1-59745-170-3_9 . 19020822 PMC3600983

[102] Zhang W, Xie M, Shu MD, Steitz JA, DiMaio D. A proximity-dependent assay for specific RNA-protein interactions in intact cells. RNA. 2016;22(11):1785–92. 10.1261/rna.058248.116 27659050 PMC5066630

[103] Mukherjee D, Coates PJ, Lorimore SA, Wright EG. Responses to ionizing radiation mediated by inflammatory mechanisms. J Pathol 2014;232(3):289–99. Epub 2013/11/21. 10.1002/path.4299 . 24254983

[104] Meziani L, Deutsch E, Mondini M. Macrophages in radiation injury: a new therapeutic target. Oncoimmunology. 2018;7(10):e1494488 Epub 2018/10/06. 10.1080/2162402X.2018.1494488 30288363 PMC6169587

[105] Zhang Y, Choksi S, Chen K, Pobezinskaya Y, Linnoila I, Liu ZG. ROS play a critical role in the differentiation of alternatively activated macrophages and the occurrence of tumor-associated macrophages. Cell Res. 2013;23(7):898–914. 10.1038/cr.2013.75 23752925 PMC3698641

[106] Rendra E, Riabov V, Mossel DM, Sevastyanova T, Harmsen MC, Kzhyshkowska J. Reactive oxygen species (ROS) in macrophage activation and function in diabetes. Immunobiology 2019;224(2):242–53. Epub 2019/02/12. 10.1016/j.imbio.2018.11.010 . 30739804

[107] McNab FW, Ewbank J, Howes A, Moreira-Teixeira L, Martirosyan A, Ghilardi N, et al Type I IFN induces IL-10 production in an IL-27-independent manner and blocks responsiveness to IFN-gamma for production of IL-12 and bacterial killing in Mycobacterium tuberculosis-infected macrophages. J Immunol. 2014;193(7):3600–12. Epub 2014/09/05. 10.4049/jimmunol.1401088 25187652 PMC4170673

[108] Howes A, Taubert C, Blankley S, Spink N, Wu X, Graham CM, et al Differential Production of Type I IFN Determines the Reciprocal Levels of IL-10 and Proinflammatory Cytokines Produced by C57BL/6 and BALB/c Macrophages. J Immunol. 2016;197(7):2838–53. Epub 2016/08/24. 10.4049/jimmunol.1501923 27549173 PMC5026030

[109] Ernst O, Glucksam-Galnoy Y, Bhatta B, Athamna M, Ben-Dror I, Glick Y, et al Exclusive Temporal Stimulation of IL-10 Expression in LPS-Stimulated Mouse Macrophages by cAMP Inducers and Type I Interferons. Frontiers in immunology. 2019;10:1788 Epub 2019/08/27. 10.3389/fimmu.2019.01788 31447835 PMC6691811

[110] Leonard JN, Ghirlando R, Askins J, Bell JK, Margulies DH, Davies DR, et al The TLR3 signaling complex forms by cooperative receptor dimerization. Proc Natl Acad Sci U S A. 2008;105(1):258–63. Epub 2008/01/04. 10.1073/pnas.0710779105 18172197 PMC2224197

[111] Mager DL, Stoye JP. Mammalian Endogenous Retroviruses. Microbiol Spectr. 2015;3(1):MDNA3-0009-2014. Epub 2015/06/25. 10.1128/microbiolspec.MDNA3-0009-2014 . 26104559

[112] Gaudray G, Gachon F, Basbous J, Biard-Piechaczyk M, Devaux C, Mesnard JM. The complementary strand of the human T-cell leukemia virus type 1 RNA genome encodes a bZIP transcription factor that down-regulates viral transcription. J Virol. 2002;76(24):12813–22. Epub 2002/11/20. 10.1128/jvi.76.24.12813-12822.2002 12438606 PMC136662

[113] Cavanagh MH, Landry S, Audet B, Arpin-Andre C, Hivin P, Pare ME, et al HTLV-I antisense transcripts initiating in the 3’LTR are alternatively spliced and polyadenylated. Retrovirology. 2006;3:15 Epub 2006/03/04. 10.1186/1742-4690-3-15 16512901 PMC1459196

[114] Barbeau B, Mesnard JM. Making sense out of antisense transcription in human T-cell lymphotropic viruses (HTLVs). Viruses. 2011;3(5):456–68. Epub 2011/10/14. 10.3390/v3050456 21994742 PMC3185765

[115] Briquet S, Richardson J, Vanhee-Brossollet C, Vaquero C. Natural antisense transcripts are detected in different cell lines and tissues of cats infected with feline immunodeficiency virus. Gene 2001;267(2):157–64. Epub 2001/04/21. 10.1016/s0378-1119(01)00404-8 . 11313142

[116] Michael NL, Vahey MT, d’Arcy L, Ehrenberg PK, Mosca JD, Rappaport J, et al Negative-strand RNA transcripts are produced in human immunodeficiency virus type 1-infected cells and patients by a novel promoter downregulated by Tat. J Virol. 1994;68(2):979–87. Epub 1994/02/01. 10.1128/JVI.68.2.979-987.1994 8289399 PMC236536

[117] Vanhee-Brossollet C, Thoreau H, Serpente N, D’Auriol L, Levy JP, Vaquero C. A natural antisense RNA derived from the HIV-1 env gene encodes a protein which is recognized by circulating antibodies of HIV+ individuals. Virology 1995;206(1):196–202. Epub 1995/01/10. 10.1016/s0042-6822(95)80034-4 . 7831774

[118] Ludwig LB, Ambrus JL Jr., Krawczyk KA, Sharma S, Brooks S, Hsiao CB, et al Human Immunodeficiency Virus-Type 1 LTR DNA contains an intrinsic gene producing antisense RNA and protein products. Retrovirology. 2006;3:80 Epub 2006/11/09. 10.1186/1742-4690-3-80 17090330 PMC1654176

[119] Barclay RA, Schwab A, DeMarino C, Akpamagbo Y, Lepene B, Kassaye S, et al Exosomes from uninfected cells activate transcription of latent HIV-1. J Biol Chem. 2017;292(36):14764 Epub 2017/09/10. 10.1074/jbc.A117.793521 28887434 PMC5592657

[120] Kolat D, Hammouz R, Bednarek AK, Pluciennik E. Exosomes as carriers transporting long noncoding RNAs: Molecular characteristics and their function in cancer (Review). Mol Med Rep. 2019;20(2):851–62. Epub 2019/06/08. 10.3892/mmr.2019.10340 31173220 PMC6625196

[121] Manghera M, Ferguson-Parry J, Lin R, Douville RN. NF-kappaB and IRF1 Induce Endogenous Retrovirus K Expression via Interferon-Stimulated Response Elements in Its 5’ Long Terminal Repeat. J Virol. 2016;90(20):9338–49. Epub 2016/08/12. 10.1128/JVI.01503-16 27512062 PMC5044829

[122] Montesion M, Williams ZH, Subramanian RP, Kuperwasser C, Coffin JM. Promoter expression of HERV-K (HML-2) provirus-derived sequences is related to LTR sequence variation and polymorphic transcription factor binding sites. Retrovirology. 2018;15(1):57 Epub 2018/08/22. 10.1186/s12977-018-0441-2 30126415 PMC6102855

[123] Schmidtmayerova H, Nuovo GJ, Bukrinsky M. Cell proliferation is not required for productive HIV-1 infection of macrophages. Virology 1997;232(2):379–84. 10.1006/viro.1997.8584 . 9191852

[124] Zhou F, Li M, Wei Y, Lin K, Lu Y, Shen J, et al Activation of HERV-K Env protein is essential for tumorigenesis and metastasis of breast cancer cells. Oncotarget. 2016;7(51):84093–117. 10.18632/oncotarget.11455 27557521 PMC5356647

[125] O’Doherty U, Swiggard WJ, Malim MH. Human immunodeficiency virus type 1 spinoculation enhances infection through virus binding. J Virol 2000;74(21):10074–80. Epub 2000/10/12. 10.1128/jvi.74.21.10074-10080.2000 . 11024136 PMC102046

[126] Ginzinger DG. Gene quantification using real-time quantitative PCR: an emerging technology hits the mainstream. Exp Hematol 2002;30(6):503–12. Epub 2002/06/14. 10.1016/s0301-472x(02)00806-8 . 12063017

[127] Schmitt K, Reichrath J, Roesch A, Meese E, Mayer J. Transcriptional profiling of human endogenous retrovirus group HERV-K(HML-2) loci in melanoma. Genome Biol Evol. 2013;5(2):307–28. Epub 2013/01/23. 10.1093/gbe/evt010 23338945 PMC3590776

