# Supplementary figures and images for: Response of human macrophages to gamma radiation is mediated via expression of endogenous retroviruses
Source: PLoS Pathog. 2021 Feb 8;17(2):e1009305. doi: 10.1371/journal.ppat.1009305 (PMC7895352; doi:10.1371/journal.ppat.1009305)

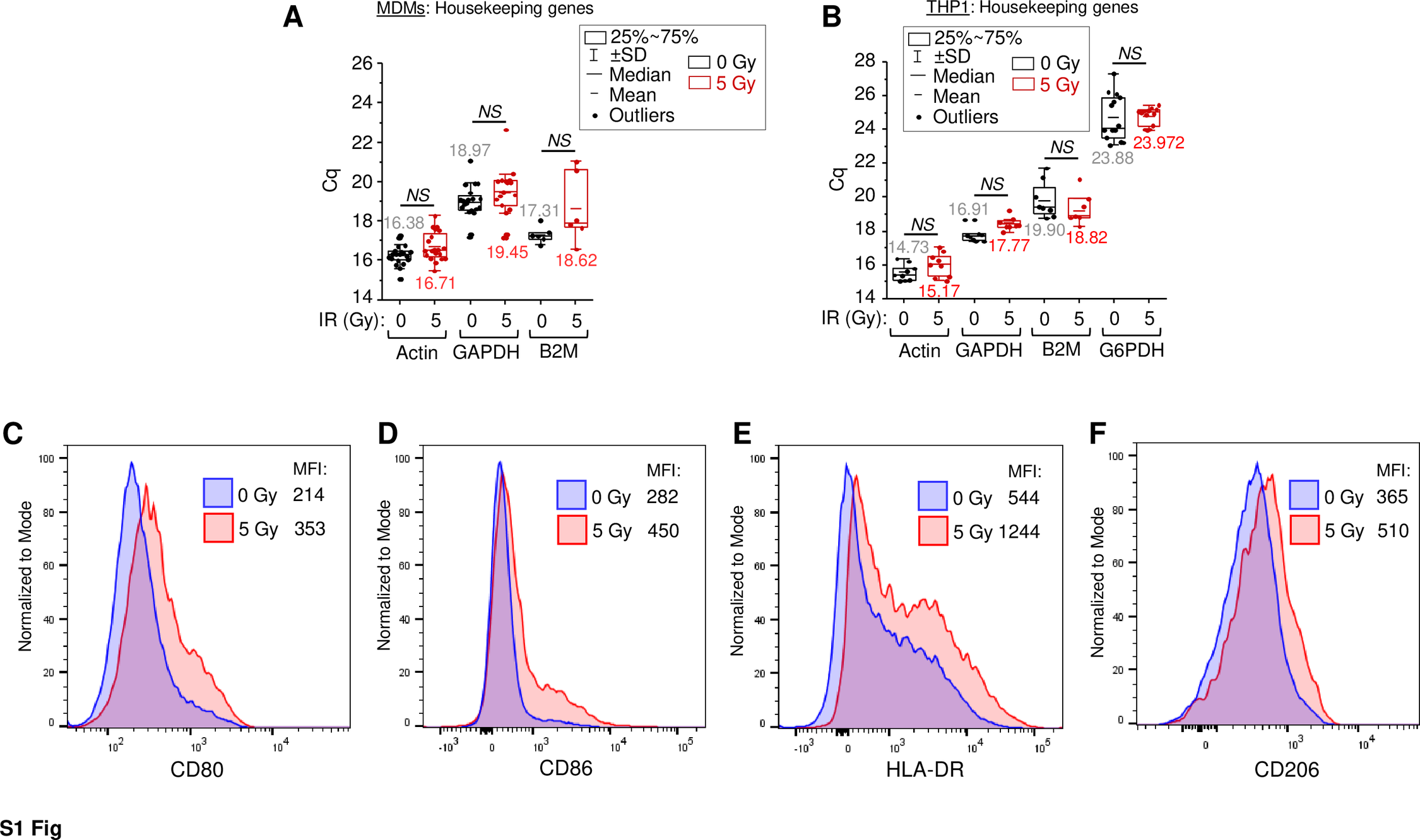

Supplement: S1 Fig — Evaluation of reference housekeeping genes for analysis of expression of macrophage activation markers (A and B); Increased expression of macrophage activation markers in the radiation-exposed THP1 cells (C-F). (A and B) RNA expression of three housekeeping genes in primary MDMs (A) and four genes in THP1 “monocytes” (B) at 48 h after exposure to 5 Gy γIR dose was assessed by RT-qPCR. Values are given as cycle threshold numbers (Cq). The boxes represent the lower and upper quartiles with lines in between representing medians; whiskers represent the range of data from three technical triplicates from minimum six biological replicates. The mean values are shown in panels; NS—p>0.05, paired Wilcoxon test. (C-E) Proportion of THP1 cells presenting inflammation surface markers CD80+ (C), CD86+ (D), HLA-DR+ (E) and anti-inflammatory marker CD206 (F) in populations of non-irradiated (0 Gy) and irradiated (5 Gy) cells detected by flow cytometry measurement of median fluorescence intensity (MFI) of viable (DAPI-) cells, 72h after γIR (graphs show representatives of 3 independent experiments). (TIF) [file ppat.1009305.s001.tif]

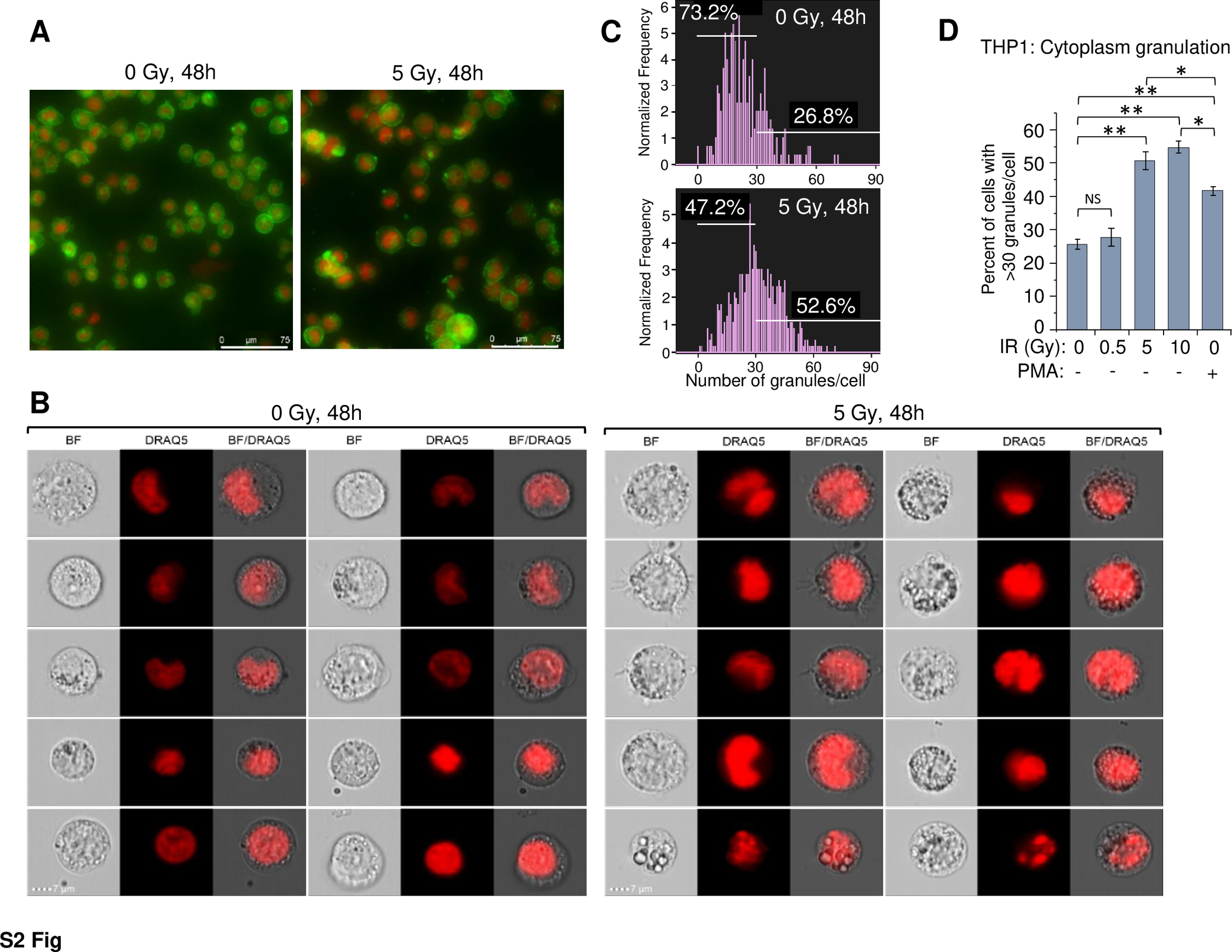

Supplement: S2 Fig — (A) Vital staining of THP1 cells with CellBrite cytoplasmic membrane dye (green) and nuclei-specific dye Draq5 (orange) to visualize size of the cytoplasm and nuclei in the control non-irradiated and 5Gy γIR exposed cells. Scale bars: 75 μm. (B) Images of irradiated or control human monocytes THP1 stained with Draq5 (nuclei), captured by imaging flow cytometry. (C) Quantitation of the effect of 5Gy γIR dose on the abundance of cytoplasmic granules in viable THP1 cells by imaging flow cytometry. Representative histograms from three biological samples are shown. (D) Quantitation of cytoplasmic granules in viable THP1 cells by imaging flow cytometry (Draq5 staining of nuclei). Error bars indicate ±SD of three independent biological replicates; * p<0.05, ** p<0.01, NS non-significant. (TIF) [file ppat.1009305.s002.tif]

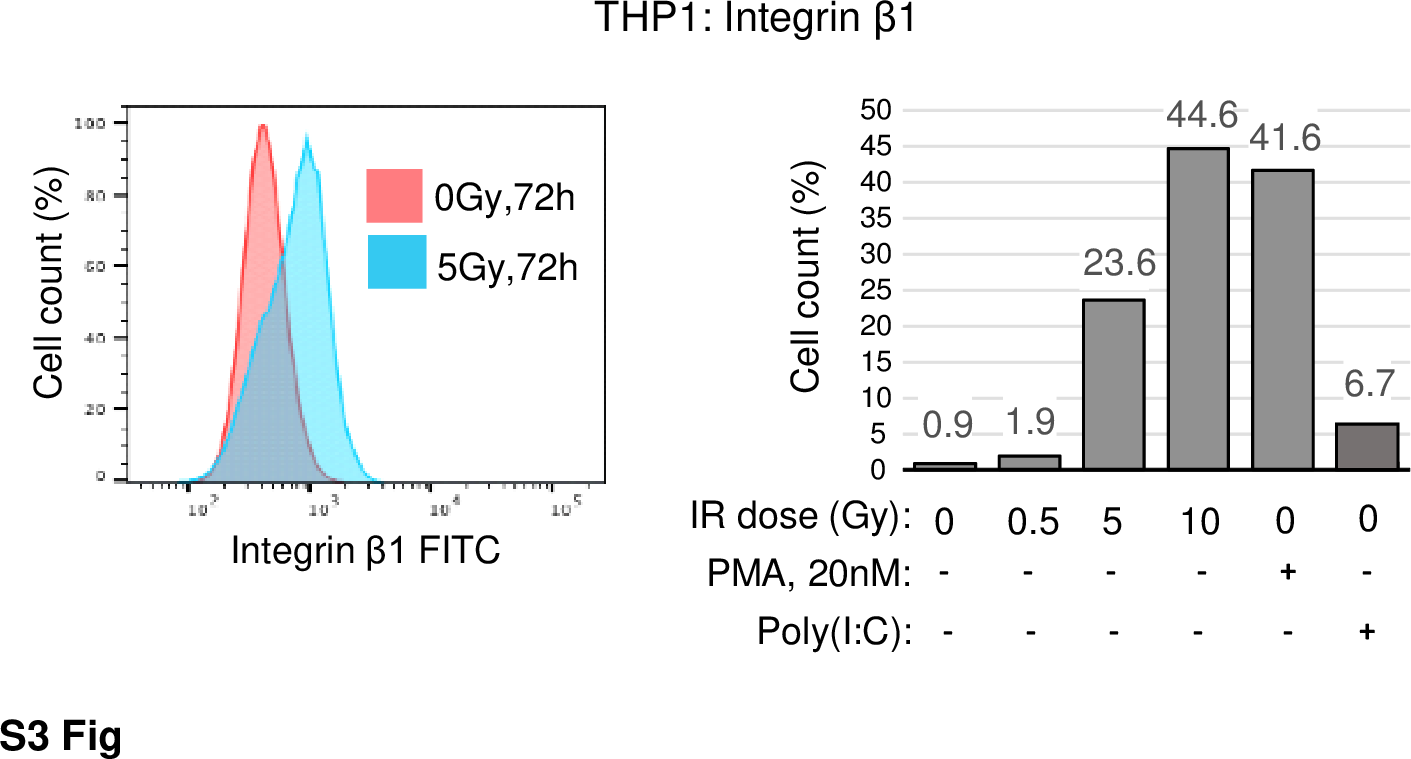

Supplement: S3 Fig — Integrin β1+ cells in populations of non-irradiated, irradiated, PMA and poly(I:C)-treated THP1 detected by flow cytometry of viable (DAPI-) cells, 72h after γIR: left panel shows ratio of integrin β1+ cells in the samples of unexposed and 5Gy dose exposed cells; the right panel shows quantitation data of integrin β1+ cells in indicated THP1 samples (flow cytometry data of viable, DAPI-negative cells were analyzed, a representative graph of three independent measurements). (TIF) [file ppat.1009305.s003.tif]

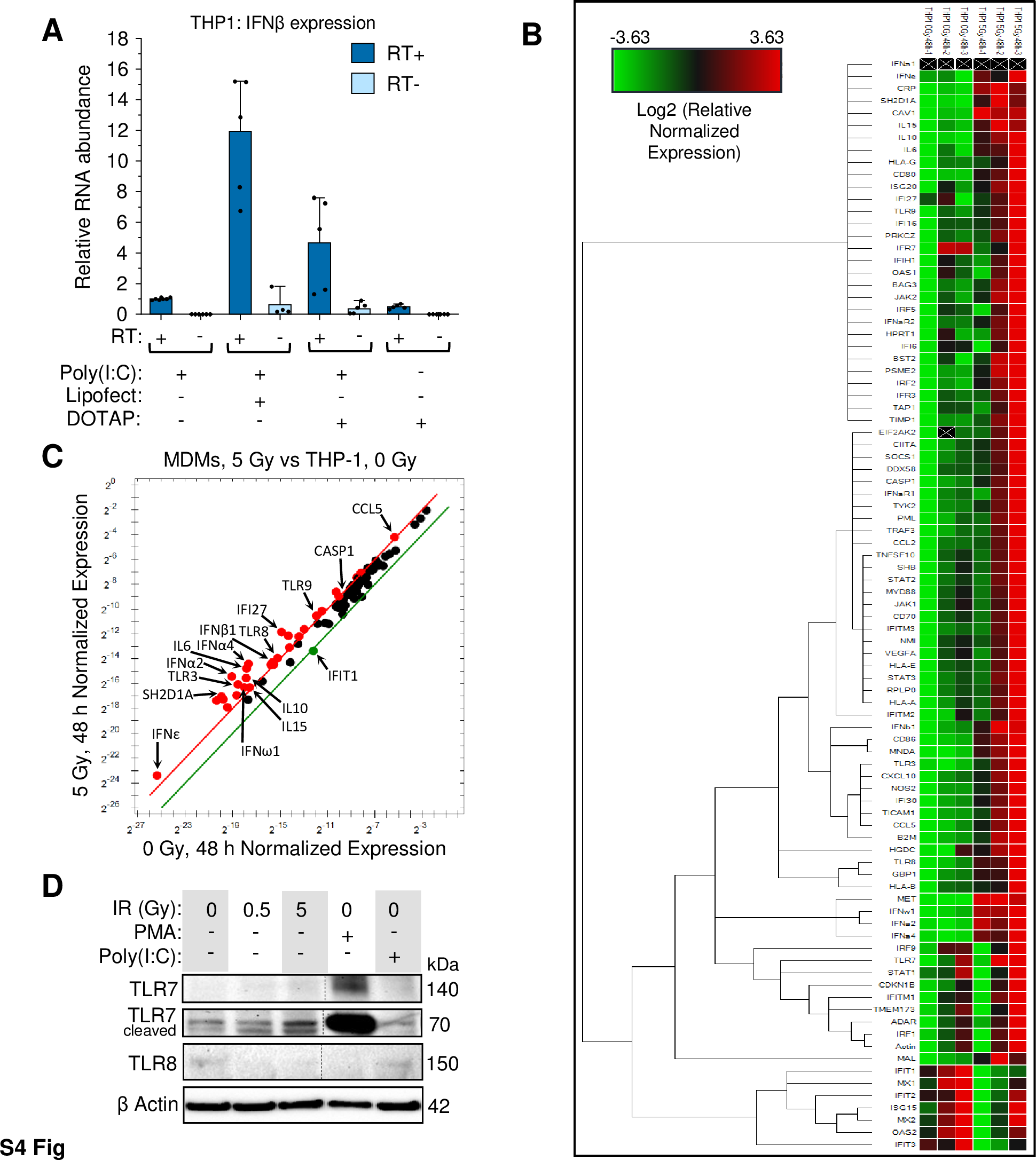

Supplement: S4 Fig — (A) Effect of dsRNA on IFNβ expression in THP1 cells, measured by RT-qPCR at 24 h after treatment. Cells were treated with poly(I:C) with or without indicated transfection reagents. Analysis of RNA abundance by RT-qPCR without reverse transcriptase reaction is shown (light-blue columns). Error bars: ±SD of 5 independent biological replicates; * p<0.05, paired Wilcoxon test. (B) Heatmap depicting expression of interferon-stimulated genes (ISG) shown in Fig 3E in THP1 cells exposed to a 5 Gy γIR dose vs. non-irradiated cells, measured by PCR array of total cellular RNA samples, 48h after γIR exposure: rows: ISG codes; columns: samples. Color codes are shown on the left panel. (C) Scatter plot showing ISG expression in irradiated vs. control MDMs, measured by PCR array of total cellular RNA samples, 48h post-irradiation. Red dots indicate twofold increase in gene expression in irradiated cells vs non-irradiated cells. (D) Western blot of cytoplasmic ssRNA sensors TLR7 and TLR8 (40 μg of total protein) in THP1 cells, 48h after exposure to indicated doses of γIR; β actin antibody served as a reference standard. (TIF) [file ppat.1009305.s004.tif]

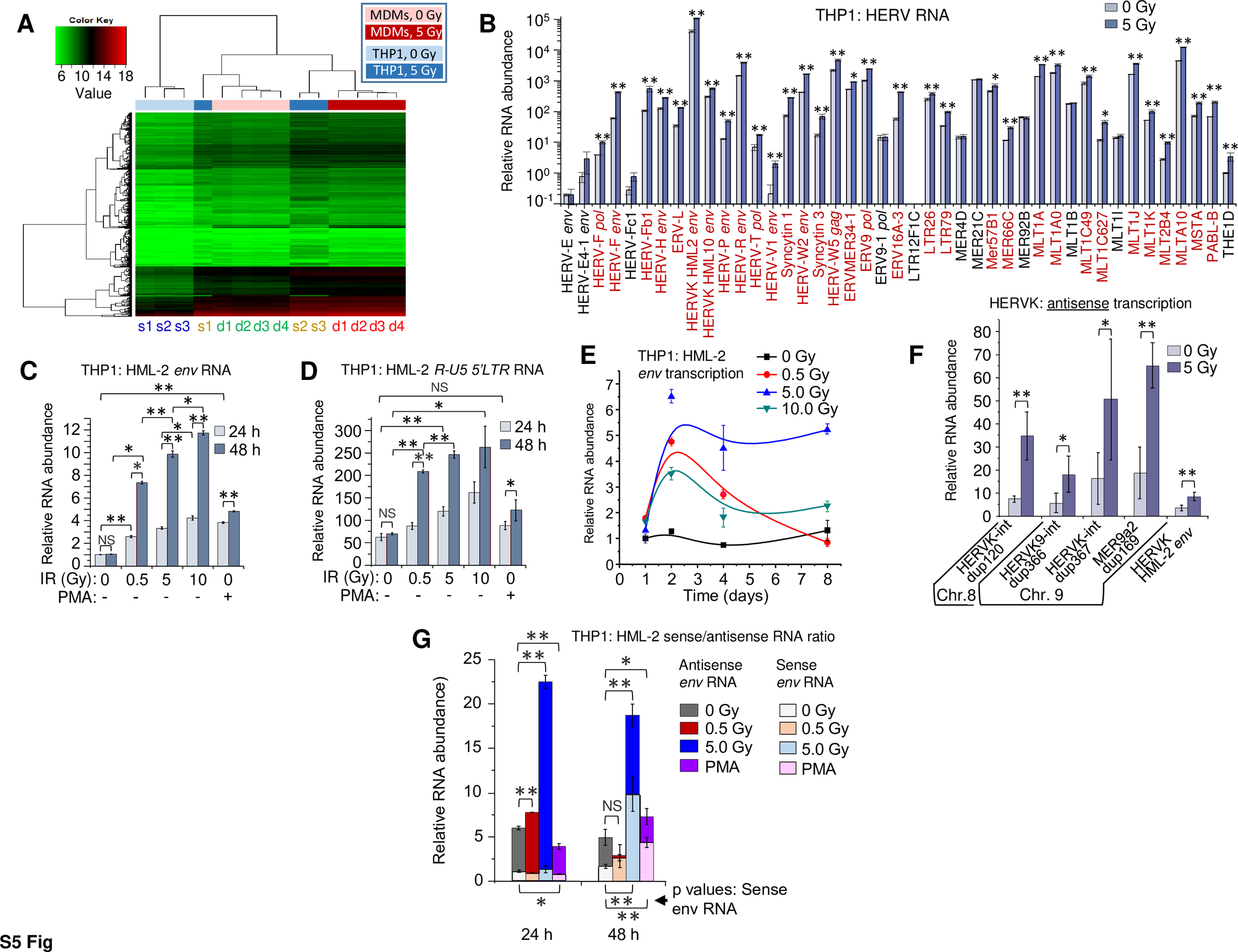

Supplement: S5 Fig — (A) Covariance-based clustered heatmap using the unique union set of 955 differentially expressed retroelements and ERVs identified by analysis of RNA-seq data across non-irradiated and irradiated THP1 (s1-s3) and MDMs (d1-d4), 48h after exposure. (B) Expression of different HERVs, identified activated in association with cancer, neurodegenerative diseases or in human embryonic cells upon differentiation [40, 42, 86–88], in THP1 cells, 48h post-irradiation. Total RNA was quantified by RT-qPCR with primers specific for 43 HERVs. Error bars: ±SD of three independent biological replicates; elements, significantly responding to γIR are indicated by red symbols. (C and D) Effect of gamma radiation doses on the transcription of HERVK HML-2 env (C) and 5’LTR (D) in THP1 cells, 48h after exposure to indicated doses of γIR, measured by RT-qPCR. The fold gene expression (ΔΔCt) was calculated in relation to β actin as a reference gene. Error bars indicate ±SD of three independent biological replicates. (E) Time course of HML-2 env expression in THP1 cells exposed to indicated doses of γIR and measured by RT-qPCR at shown time-points. B-spline curves were plotted based on the values of fold gene expression for each time point calculated as ΔΔCt relative to β-actin. Error bars: ±SD of four independent biological replicates. (F) Relative count of antisense RNA, measured by RT-qPCR, of differentially expressed HERVK (sense RNA count shown in Fig 4C) in THP1 cells, 48h post-irradiation. Error bars: ±SD of three independent biological replicates. (G) Gamma radiation activates antisense transcription of HERVK HML-2 earlier than the sense transcription: relative RNA count, measured by RT-qPCR, of HML-2 sense and antisense env transcripts, 24 and 48h after irradiation of THP1 cells. In all panels, * p<0.05, ** p<0.01, NS non significant. (TIF) [file ppat.1009305.s005.tif]

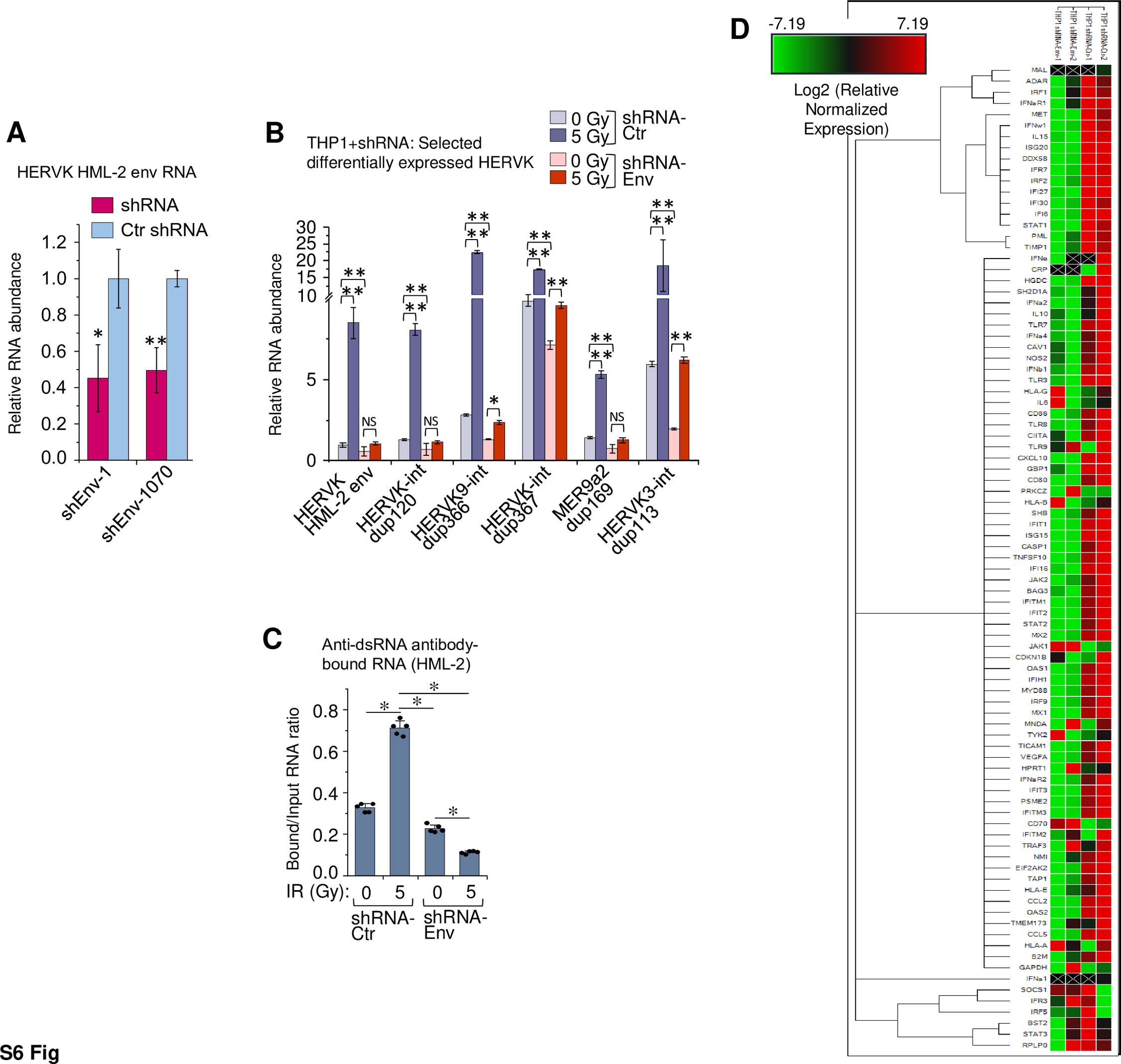

Supplement: S6 Fig — (A) Relative count of HERVK HML-2 env RNA in THP1 cells infected with lentivial vector pLKO.1 puro expressing indicated shRNA, selected with 0.5 μg/ml puromycin. RNA was isolated and relative env RNA abundance was measured by RT-qPCR. The fold change RNA count (ΔΔCt) was calculated in relation to β actin reference gene. Error bars indicate ±SD of three independent biological replicates. (B) Transcription, measured by RT-qPCR, of randomly selected differentially expressed HERVK proviruses, identified by transcriptomic analysis, in THP1 cells expressing control (grey) or shRNA-Env (red), 48h post-irradiation. In panels A and B, error bars: ±SD of three independent biological replicates; * p<0.05, ** p<0.01, two-tailed paired t test. (C) Ratio of HML-2 RNA bound to anti-dsRNA antibodies to RNA input. RT-qPCR of RNA IP complexes with rJ2 and 9D5 antibodies, 48h post-irradiation. Error bars: ±SD of five independent biological replicates; * p<0.05, paired Wilcoxon test. (D) Heatmap depicting expression of interferon-stimulated genes (ISG) shown on Fig 5E in THP1 cells exposed to 5Gy γIR, 48h post-exposure: cells expressing shRNA-Env vs control shRNA, measured by PCR array of total cellular RNA samples. Rows: ISG codes; columns: samples. Color codes are shown on the left panel. (TIF) [file ppat.1009305.s006.tif]
